# Two apicoplast dwelling glycolytic enzymes provide key substrates for metabolic pathways in the apicoplast and are critical for *Toxoplasma* growth

**DOI:** 10.1371/journal.ppat.1011009

**Published:** 2022-11-30

**Authors:** Zhipeng Niu, Shu Ye, Jiaojiao Liu, Mengyu Lyu, Lilan Xue, Muxiao Li, Congcong Lyu, Junlong Zhao, Bang Shen

**Affiliations:** 1 State Key Laboratory of Agricultural Microbiology, College of Veterinary Medicine, Huazhong Agricultural University, Wuhan, Hubei Province, PR China; 2 Key Laboratory of Preventive Veterinary Medicine in Hubei Province, Wuhan, Hubei Province, PR China; 3 Hubei Hongshan Laboratory, Wuhan, Hubei Province, PR China; 4 Shenzhen Institute of Nutrition and Health, Huazhong Agricultural University, Shenzhen, Guangdong Province, PR China; 5 Shenzhen Branch, Guangdong Laboratory for Lingnan Modern Agriculture, Genome Analysis Laboratory of the Ministry of Agriculture, Agricultural Genomics Institute at Shenzhen, Chinese Academy of Agricultural Sciences, Shenzhen, Guangdong Province, PR China; University of Geneva, SWITZERLAND

## Abstract

Many apicomplexan parasites harbor a non-photosynthetic plastid called the apicoplast, which hosts important metabolic pathways like the methylerythritol 4-phosphate (MEP) pathway that synthesizes isoprenoid precursors. Yet many details in apicoplast metabolism are not well understood. In this study, we examined the physiological roles of four glycolytic enzymes in the apicoplast of *Toxoplasma gondii*. Many glycolytic enzymes in *T*. *gondii* have two or more isoforms. Endogenous tagging each of these enzymes found that four of them were localized to the apicoplast, including pyruvate kinase2 (PYK2), phosphoglycerate kinase 2 (PGK2), triosephosphate isomerase 2 (TPI2) and phosphoglyceraldehyde dehydrogenase 2 (GAPDH2). The ATP generating enzymes PYK2 and PGK2 were thought to be the main energy source of the apicoplast. Surprisingly, deleting PYK2 and PGK2 individually or simultaneously did not cause major defects on parasite growth or virulence. In contrast, TPI2 and GAPDH2 are critical for tachyzoite proliferation. Conditional depletion of TPI2 caused significant reduction in the levels of MEP pathway intermediates and led to parasite growth arrest. Reconstitution of another isoprenoid precursor synthesis pathway called the mevalonate pathway in the TPI2 depletion mutant partially rescued its growth defects. Similarly, knocking down the GAPDH2 enzyme that produces NADPH also reduced isoprenoid precursor synthesis through the MEP pathway and inhibited parasite proliferation. In addition, it reduced *de novo* fatty acid synthesis in the apicoplast. Together, these data suggest a model that the apicoplast dwelling TPI2 provides carbon source for the synthesis of isoprenoid precursor, whereas GAPDH2 supplies reducing power for pathways like MEP, fatty acid synthesis and ferredoxin redox system in *T*. *gondii*. As such, both enzymes are critical for parasite growth and serve as potential targets for anti-toxoplasmic intervention designs. On the other hand, the dispensability of PYK2 and PGK2 suggest additional sources for energy in the apicoplast, which deserves further investigation.

## Author summary

The apicoplast is a unique organelle in most apicomplexan parasites, a large group of unicellular pathogens that infect humans and animals. It is essential for parasite growth as it hosts important metabolic pathways and provides critical metabolites. By endogenous gene tagging, here we found four glycolytic isoenzymes localized to the apicoplast of *Toxoplasma gondii*, including PYK2, PGK2, TPI2 and GAPDH2. Gene inactivation studies demonstrated that the two ATP generating enzymes PYK2 and PGK2 were dispensable for the growth of *T*. *gondii*, whereas TPI2 and GAPDH2 were essential. Combined genetic and metabolic analyses suggest that TPI2 generated glyceraldehyde-3-phosphate is critical for the MEP pathway to synthesize isoprenoid precursors, which are essential for parasites proliferation. On the other hand, GAPDH2 is probably a main source of reducing power for metabolic enzymes involved in isoprenoid precursor and fatty acid synthesis in the apicoplast. As such, both enzymes are required for optimal parasite growth.

## Introduction

*Toxoplasma gondii* is an obligate intracellular pathogen belonging to the phylum Apicomplexan, which contains many parasitic organisms that are of great medical and veterinary concerns, such as *Plasmodium* spp. that cause malaria and *Cryptosporidium* spp. that cause diarrhea in humans and animals [1,2]. *T*. *gondii* is capable of infecting all nucleated cells and proliferates rapidly as tachyzoites in host cells within a membrane bound structure called the parasitophorous vacuole (PV). The nonfusogenic nature of the PV provides a safe environment for the parasites to replicate. On the other hand, the membrane surrounding it forms a barrier for the parasites to obtain nutrients from host cells freely. As a result, *T*. *gondii* encodes proteins like GRA17 and GRA23 to form pores on the PV membrane to allow entry of small metabolites into the PV [3]. Although equipped with such nutrient scavenging machineries, the parasites can not acquire all necessary metabolites from host cells. In fact, they have complex metabolic capacities that allow the parasites to establish parasitism in diverse environments. Published work has demonstrated great metabolic plasticity of *T*. *gondii* tachyzoites, being able to use glucose, glutamine and even lactate and amino acids as carbon sources [4–6]. Genome-scale metabolic modeling, along with CRISPR-Cas9 based genetic screen on all known metabolic genes further illustrated the metabolic flexibility of this organism [7].

Glycolysis is an ancient metabolic pathway that is active in almost all cells. Although extensively studied, the roles of glycolysis under different physiological and pathological conditions are still not fully understood. *T*. *gondii* encodes a full set of glycolytic genes but we started to appreciate their functions only recently. Inactivation of the first enzyme hexokinase (HK) only modestly affected tachyzoite growth, but it drastically reduced mature cyst formation *in vivo* [8]. Similar to the mutant lacking the major glucose transporter GT1 [5], the growth of *Δhk* tachyzoites was supported by glutaminolysis, which not only supplied ATP through the TCA cycle, but also provided glycolytic intermediates through phosphoenolpyruvate carboxykinase (PEPCK) and gluconeogenesis [9]. Conditional depletion of fructose-1,6-bisphosphate aldolase (ALD) did not cause significant growth or invasion defects in a limited time period [10]. On the other hand, two glycolytic enzymes, the pyrophosphate-dependent phosphofructokinase (PFK2) and the pyruvate kinase 1 (PYK1), were shown to be critical for tachyzoite growth [11,12]. The former is thought to have a regulatory role to achieve balanced catabolism and anabolism in the parasite, through the coupling of pyrophosphate (product of many anabolic reactions) hydrolysis to glycolysis. PYK1 is the enzyme mainly responsible for pyruvate production in the cytoplasm. Pyruvate is a key metabolite for central carbon metabolism and it is involved in multiple metabolic pathways, such as fatty acids synthesis, TCA cycle and the methylerythritol phosphate (MEP) pathway that synthesizes isoprenoid precursors [12]. In addition, the gluconeogenic enzyme fructose 1,6-bisphosphatase 2 (FBPase2) was found to be essential for parasite growth even in the presence of glucose [13]. FBPase2 depletion results in global alteration of carbon metabolism, including catabolism like glycolysis and TCA cycle, as well as anabolism like fatty acids, glycolipids, and amylopectin synthesis. It was proposed that FBPase2 might mediate futile cycling between gluconeogenesis and glycolysis that allows the parasites to rapidly adapt to changing environments. Altogether, these studies show that glycolysis is indeed critical for the lytic cycle of *T*. *gondii* tachyzoites, although certain steps are dispensable due to carbon source flexibility.

One interesting aspect of the glycolytic pathway in *T*. *gondii* is that most glycolytic enzymes have two or more isoforms. Those different isoforms have distinct expression patters during the parasite’s life cycle, or have different subcellular localization or enzymatic properties [13]. A number of glycolytic isoenzymes display stage specific expression. Enolase 2 (ENO2) and lactate dehydrogenase 1 (LDH1) are mainly produced in tachyzoites, whereas ENO1 and LDH2 proteins are predominantly expressed in bradyzoites during asexual growth [14]. The physiological significance of such stage specific expression is not fully understood yet. Both LDH1 and LDH2 are dispensable for tachyzoite growth under standard culture conditions with high levels of oxygen. However, LDH1 is critical for parasite propagation *in vivo* or under hypoxia conditions. As such, mutant lacking LDH1 may be a good live attenuated vaccine candidate [15]. Besides expression patterns, some isoenzymes show different catalytic properties. For example, while the above mentioned PFK2 is a pyrophosphate-dependent phosphofructokinase, *T*. *gondii* also encodes a canonical ATP-dependent phosphofructokinase [11]. But genetic studies found that it was not required for tachyzoite growth. In addition, Fleige *et al*. reported the localization of three glycolytic enzymes into the apicoplast, including TPI2 (triosephosphate isomerase 2), PGK2, and PYK2 [16]. Localization of glycolytic enzymes into the apicoplast was also reported or predicted in other apicomplexan parasites, such as TPI and PYK2 in *Plasmodium spp* [17–19]. The biological significance of many such enzymes in the apicoplast has not been clearly defined. Existing studies on PYK2 in *T*. *gondii* and *Plasmodium spp* indicate that its roles can be different in these two organisms, being essential in *Plasmodium spp* but dispensable in *T*. *gondii* [12,19].

The apicoplast is a relict plastid found in many apicomplexan parasites that is homologous to the chloroplasts of algae and plants [20]. It is derived from secondary endosymbiosis with a cyanobacterial origin, but the photosynthetic activity is lost [20,21]. On the other hand, it retains many hallmarks of its ancestry like a circular genome and homes a number of biosynthetic pathways that are distinct from those in host cells. These include the MEP pathway that uses pyruvate and 3-glyceraldehyde phosphate as substrates to produce isopentenyl diphosphate (IPP) and its isomer dimethylallyl diphosphate (DMAPP), two essential precursors for the synthesis of all isoprenoids [22,23]. Human and animal hosts use a completely different pathway called the mevalonate pathway that is in the cytoplasm to synthesize IPP and DMAPP [24]. Because isoprenoids are essential for almost all cells due to their diverse roles from protein modification to structure maintenance, the MEP pathway is believed to be a great target for anti-parasitic drug design [25,26]. Indeed, the antibiotic fosmidomycin that targets the DOXP reductoisomerase (DOXPRI, also called DXR or IspC) in the MEP pathway is an effective antimalaria drug [27,28]. Genetic ablation of DOXPRI and 4-hydroxy-3-methylbut-2-enyl diphosphate (HMBPP) reductase (called IspH or LytB) in *T*. *gondii* also demonstrated the essentiality of the MEP pathway [23]. The type II fatty acid synthesis pathway FASII is another well studied metabolic pathway in the apicoplast. It is involved in the *de novo* synthesis of long-chain fatty acids, mainly myristic acid and palmitic acid. Like the MEP pathway, mammalian hosts also do not have the FASII pathway. For a long time, FASII was also thought to be a good drug target [29–32]. Nonetheless, gene deletion studies on FASII enzymes like FabI, FabD and FabZ demonstrated that this pathway is not essential for the growth of blood stage *Plasmodium spp* or tachyzoite stage *T*. *gondii* [33–35]. In addition, *T*. *gondii* encodes an apicoplast localized pyruvate dehydrogenase complex (PDH), which generates acetyl-CoA to fuel FASII. Deleting any of the PDH subunits only modestly reduced parasite proliferation and did not affect parasite virulence, further confirming the dispensability of FASII. This is explained by parasite’s ability to salvage fatty acids from the environments [36]. The apicoplast also contains other pathways like heme biosynthesis and iron-sulfur cluster biosynthesis, both of which are critical for *T*. *gondii* growth [37,38]. Isoprenoid precursor synthesis through the MEP pathway was thought to be the only essential role of the apicoplast in *Plasmodium* parasites, since IPP supplementation could rescue the growth of parasites devoid of apicoplasts [39]. However, recently it was shown that in the absence of intact apicoplasts, the apicoplast enzyme dephospho-CoA kinase (DPCK) involved in coenzyme A synthesis was found to localize to vesicles. More importantly, DPCK was found to be essential even in MEP bypass lines without apicoplasts, suggesting that the vesicles can host essential functions after apicoplast disruption in blood-stage *P*. *falciparum* parasites [40].

The current model of metabolism in *T*. *gondii* apicoplast suggests that the apicoplast phosphate translocator (APT) imports glycolytic intermediates from the cytosol, which are then further utilized by downstream glycolytic enzymes in the apicoplast [41]. However, the exact biological significant of each of these apicoplast localized enzymes are not fully understood. In this study, we analyzed the roles of four such enzymes in *T*. *gondii*. The results show that TPI2 and GAPDH2 are critical for parasite growth, whereas PGK2 and PYK2 are not. With these data, we have provided an improved model for metabolism in *T*. *gondii* apicoplast.

## Results

### Four glycolytic enzymes localized to the apicoplast of *T*. *gondii*

**Fig 1:**
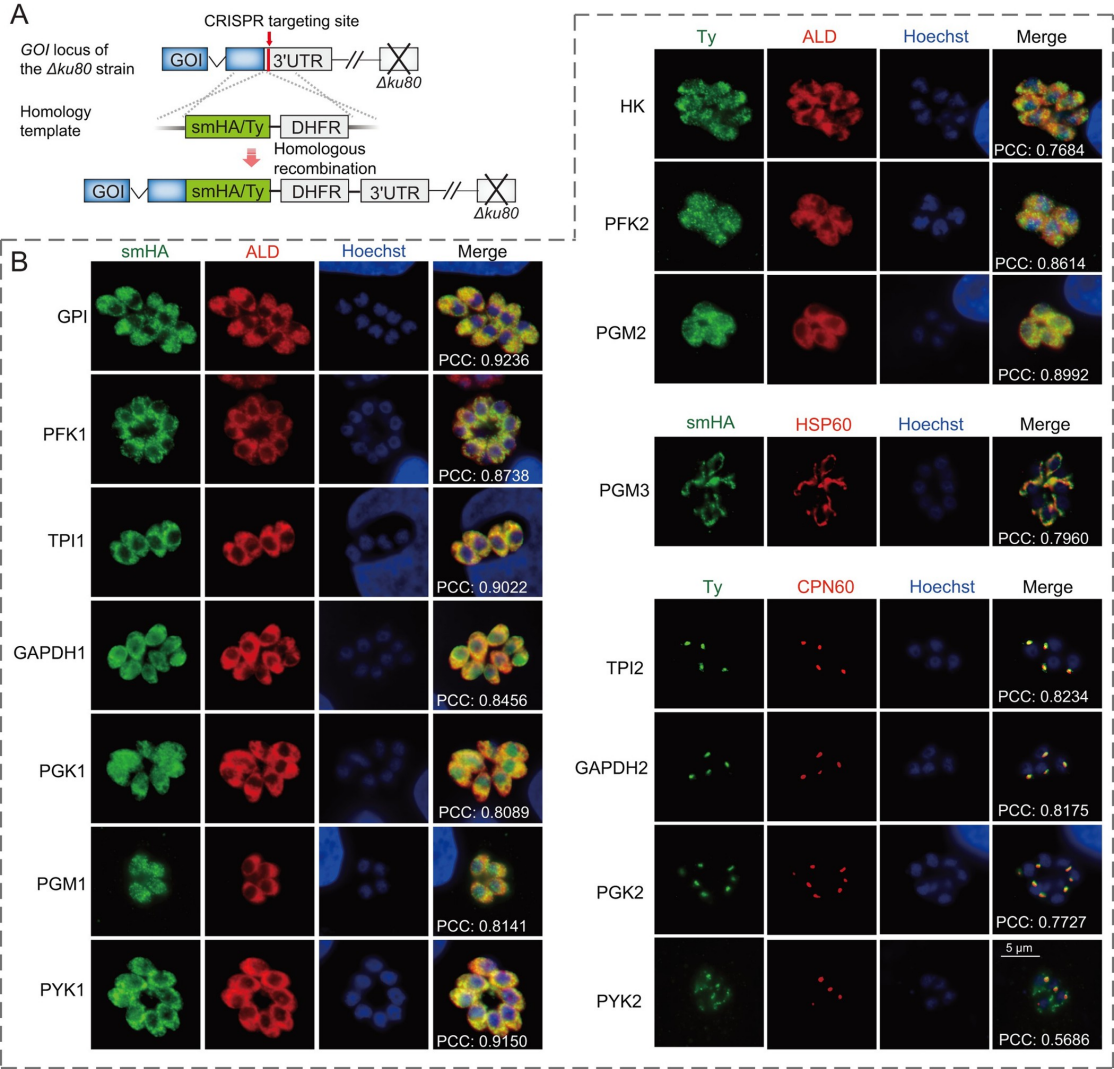
Subcellular localization patterns of glycolytic isoenzymes in *T*. *gondii* tachyzoites. A, strategy for C terminal tagging of a gene of interest (GOI) with an smHA or Ty tag at the endogenous locus, through CRISPR/Cas9 mediated site specific integration in the *Δku80* strain. DHFR is the selection marker that confers pyrimethamine resistance. B, Subcellular localization of selected glycolytic isoenzymes, determined by immunofluorescent staining of corresponding tagged strains generated in A. ALD, HSP60 and CPN60 were used as cytosol, mitochondrion, and apicoplast specific markers, respectively. The degree of co-localization between tagged proteins and organelle specific markers were quantified by Pearson’s correlation and expressed as Pearson correlation coefficient (PCC).

Through ectopic expression of epitope tagged genes, three glycolytic isoenzymes have been shown to localize to the apicoplast of *T*. *gondii* tachyzoites [16]. To further check the subcellular localization of glycolytic enzymes, each encoding gene was tagged with an smHA or Ty tag at the endogenous locus and the corresponding transgenic line was examined by immunofluorescent microscopy (Fig 1A). Of the 19 glycolytic enzymes predicted, 15 (except the two aldolases and two enolases that have been well characterized [42–44]) were analyzed by this approach and 10 were found to localize to the parasite cytoplasm with co-localization with aldolase 1 (ALD) (Fig 1B). Four enzymes, including TPI2, GAPDH2, PGK2 and PYK2, displayed clear apicoplast localization, as evident by co-localization with the apicoplast marker CPN60 (Fig 1B). PYK2 also showed weak localization outside the apicoplast, consistent with the mitochondrion and apicoplast dual localization reported before [45]. Interestingly, we found three putative phosphoglycerate mutases (PGMs) in the *T*. *gondii* genome, two of which (PGM1: TGME49_273030 and PGM2: TGME49_297060) were described before [16]. Our endogenous gene tagging found that both PGM1 and PGM2 were localized to the parasite cytosol (Fig 1B). The third potential PGM enzyme PGM3 (TGME49_222910) was localized to the mitochondrion (Fig 1B). The sequence conservation among these three PGMs are very low and their enzymatic activities have not been verified. As such, whether they are *bona fide* PGMs needs further confirmation. Nevertheless, these results show that some glycolytic isoenzymes do localize to subcellular compartments other than the cytosol, which is consistent with what was reported previously [16].

### The two energy generating enzymes in the apicoplast are dispensable for parasite growth

**Fig 2:**
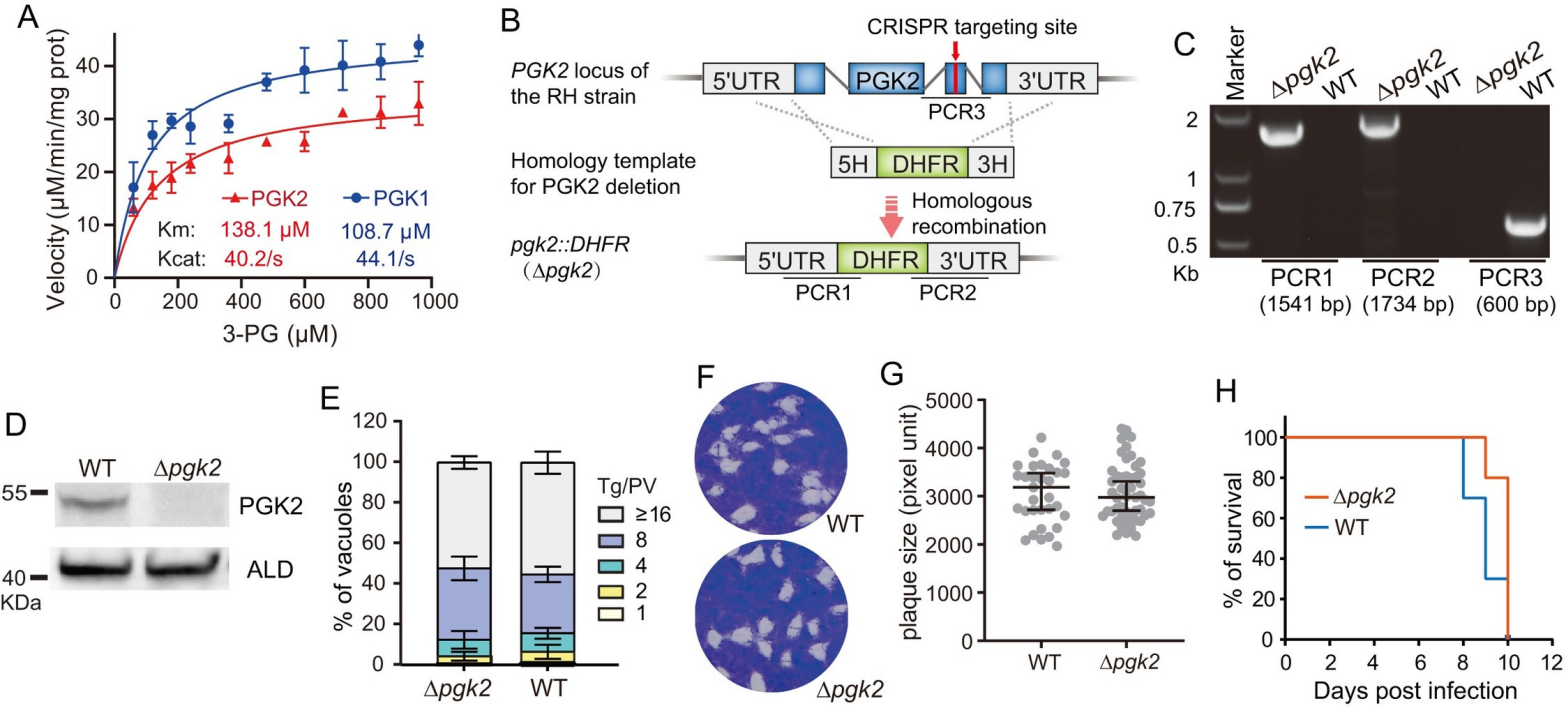
PGK2 is dispensable for *T*. *gondii* growth. A, comparison of the enzymatic activities of *T*. *gondii* PGK1 and PGK2, assayed with recombinant enzymes under different concentrations of 3-phosphoglycerate (3-PG). Means **± SD of three independent experiments were plotted. B, schematic illustration of *PGK2* deletion by** CRISPR/Cas9 directed homologous gene replacement with the selection marker *DHFR*. **5H and 3H denote the two homologous arms for recombination. PCR1/2/3 are amplification products for *PGK2* deletion mutant (*Δpgk2*) identification. C, diagnostic PCR on a *Δpgk2* clone. D, Western blotting confirms the absence of PGK2 expression in the *Δpgk2* mutant. ALD was included as a loading control. E, comparison of the intracellular replication rates of the wildtype (WT) and the *Δpgk2* mutant, as determined by the distribution of the number (1, 2, 4, 8, ≥16) of *Toxoplasma* parasite per PV (Tg/PV).** Means **± SEM of three independent experiments, each with three replicates. More than 120 PVs were analyzed for each strain in each experiment. F, plaque formation of WT and *Δpgk2*** parasites on HFF monolayers after 200 tachyzoites of each strain were used to infect HFF cells for 7 days. G, sizes of plaques derived from F, as expressed in pixel units when measured by Adobe Photoshop. H, survival curves of mice infected with **WT or *Δpgk2*** parasites. Each strain was tested by 10 mice.

PYK2 is localized to the apicoplast in both *T*. *gondii* and *Plasmodium spp* and was thought to be the main source for ATP production in this organelle. PYK2 is indeed essential in *P*. *falciparum* and it was shown to be critical for the maintenance of the apicoplast by providing NTP and dNTP, which contribute to apicoplast genome replication, transcription, and other biosynthetic activities [19]. Nonetheless, disruption of PYK2 (phenotype score derived from a genome-wide CRISPR screen [46]: -2.76) in *T*. *gondii* did not affect tachyzoite growth, suggesting additional energy sources in the *T*. *gondii* apicoplast [12]. The protein localization results shown above suggest that PGK2 may be an alternative energy source in the apicoplast. Phosphoglycerokinases catalyze the conversion of 1,3-bisphosphoglycerate (1,3-PG) to 3-phosphoglycerate (3-PG), along with ATP production. To check whether PGK2 contributes to energy supply in the apicoplast, we first determined the enzymatic activity of PGK2 and compared it to that of the cytosolic PGK1. Both enzymes were expressed in and purified from *E*. *coli* as recombinant proteins (S1 Fig). Subsequently their enzymatic activity was determined by an NADH coupled colorimetric assay under various 3-PG concentrations (assayed in the reverse direction of the reaction catalyzed by PGK). The results show that both PGKs are active enzymes, with the activity of PGK1 being slightly higher than that of PGK2 (Fig 2A).

To check the biological function of PGK2 during parasite growth, it was subjected to gene deletion analyses. Using a CRISPR/CAS9 mediated homologous gene replacement strategy (Fig 2B), PGK2 was completely knocked out in the type 1 strain RH and replaced by the pyrimethamine-resistant cassette *DHFR* (dihydrofolate reductase). Diagnostic PCRs were performed to verify the replacement of PGK2 in *Δpgk2* mutants (Fig 2C). Western blotting further confirmed the disruption of PGK2 as no specific product could be detected in the mutant using a polyclonal antibody against the PGK2 protein (Fig 2D). Successful deletion of PGK2 suggests that it is not essential for parasite growth or survival, which contrasts with the relatively low phenotype score (-3.58) of PGK2 derived from a genome-wide loss of function screen [46]. Intracellular replication and plaque formation assays *in vitro* further demonstrated that PGK2 is dispensable for the growth of tachyzoites, as the proliferation rates and plaquing efficiency of the *Δpgk2* mutant were indistinguishable from that of the parental strain (Fig 2F and 2G). In addition, the virulence of the *Δpgk2* mutant in mice is also similar to that of the wildtype strain (Fig 2H). Taken together, these results show that although the apicoplast localized PGK2 is an active PGK, it is not the main source of energy in *T*. *gondii* apicoplast.

**Fig 3:**
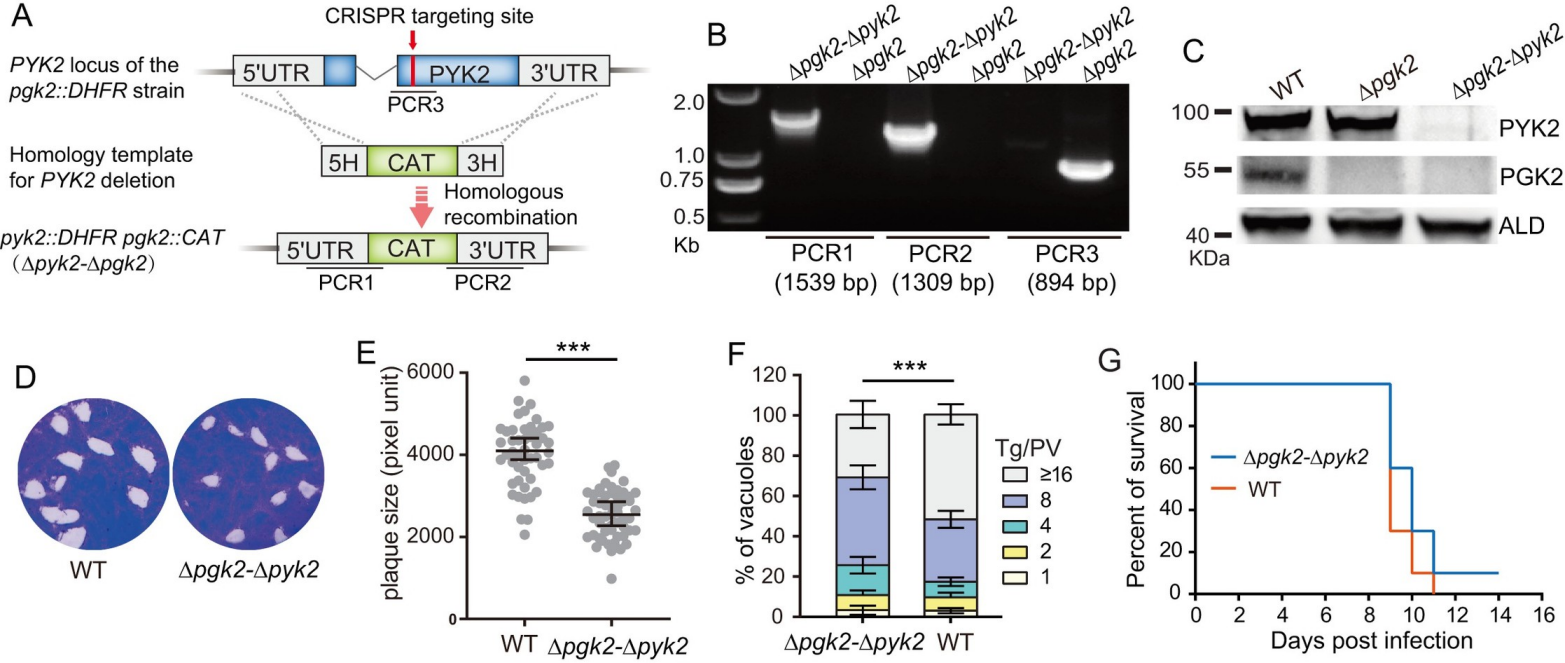
The *Δpgk2-Δpyk2* mutant is viable *in vitro* and virulent *in vivo*. A, strategy for the construction of the ***Δpgk2-Δpyk2* mutant, which was generated by replacing PYK2 in the *Δpgk2* mutant with the chloramphenicol resistant marker CAT. B, diagnostic PCR on one *Δpgk2-Δpyk2* clone. C, Western blotting examining the expression of PGK2 and PYK2 in indicated strains, using antibodies against PGK2 and PYK2 respectively. ALD was included as a loading control. D, a 7-day plaque assay comparing the overall growth of the *Δpgk2-Δpyk2*** mutant to that of WT parasites. E, relative sizes of plaques. Means **± SEM of over 90 plaques,** ***P < 0.001, student’s t-test. F, intracellular proliferation **rates of the *Δpgk2-Δpyk2*** mutant vs the WT strain RH, as determined by replication assay described in Fig 2E. Means **± SEM of three independent experiments, each with three replicates.** ***P < 0.001, two-way ANOVA followed by Tukey’s multiple comparison tests. G, survival curves of mice infected with indicated strains, each was tested by 10 mice.

Both PGK2 and PYK2 are ATP-yielding enzymes in the apicoplast. As such, there might be functional redundancy between these two genes. To test this possibility, we knocked out *PYK2* in the *Δpgk2* mutant to generate a double deletion mutant *Δpgk2-Δpyk2* (Fig 3A). Diagnostic PCRs confirmed the complete replacement of *PYK2* by the chloramphenicol resistant marker *CAT* in the double mutant (Fig 3B). Western blotting using antibodies against PYK2 and PGK2 proteins further assured the loss of expression of these two proteins in the double mutant (Fig 3C). When plaque assays were used to assess the overall fitness of parasites, it was found that the *Δpgk2-Δpyk2* mutant formed smaller plaques than the wildtype strain RH (Fig 3D and 3E). Replication assays also reported a slightly reduced proliferation rate of the *Δpgk2-Δpyk2* mutant (Fig 3F). However, when used to infect mice, these two strains displayed very similar survival plots, indicating that simultaneous inactivation of PGK2 and PYK2 had no influence on acute virulence of the parasites (Fig 3G). These results imply that there are other ways to provide energy for the apicoplast besides PYK2 and PGK2.

In contrast to the dispensability of PYK2, the cytosolic PYK1 (phenotype score: -4.52) was shown to be crucial for tachyzoite growth [12]. To check whether the same situations happen to the PGK enzymes, the cytosolic PGK1 was subjected to gene deletion studies. Direct deletion using the same approach described in Figs 2B and 3A failed to generate a clonal line of *Δpgk1*, suggesting that it might have indispensable roles. To further check this possibility, an inducible knockdown strain (iPGK1) was constructed to deplete PGK1 expression in the presence of anhydrotetracycline (ATc) (S2A Fig). In the iPGK1 strain, the endogenous promoter of PGK1 was replaced by a tetracycline regulatable promoter (pS1O7) and the N terminal of PGK1 was fused with a Ty tag (S2A Fig). Diagnostic PCRs confirmed the correct promoter replacement in iPGK1 and Western blotting demonstrated the depletion of PGK1 expression to nondetectable level after 48 hours ATc treatment (S2B and S2C Fig). Subsequent phenotypic analyses indicate PGK1 is indeed critical for optimal growth of tachyzoites. Shutdown of PGK1 expression significantly reduced plaque development in HFF monolayers (S2D and S2E Fig) and suppressed parasite replication within host cells (S2F Fig). Interestingly, the decreased replication observed in the *PGK1* depletion mutant was probably caused by the accumulation of toxic glycolytic intermediates after PGK1 inactivation, because without glucose in the culture medium the iPGK1 strain had similar replication rates with or without ATc treatment, like that in the parental strain TATi (S2F Fig). These results suggest that while the apicoplast localized PGK2 is dispensable, the cytosolic PGK1 is required for efficient utilization of glucose to support robust tachyzoite propagation.

### Both the cytosol and apicoplast localized triose-phosphate isomerases are needed for the lytic cycle of tachyzoites

**Fig 4:**
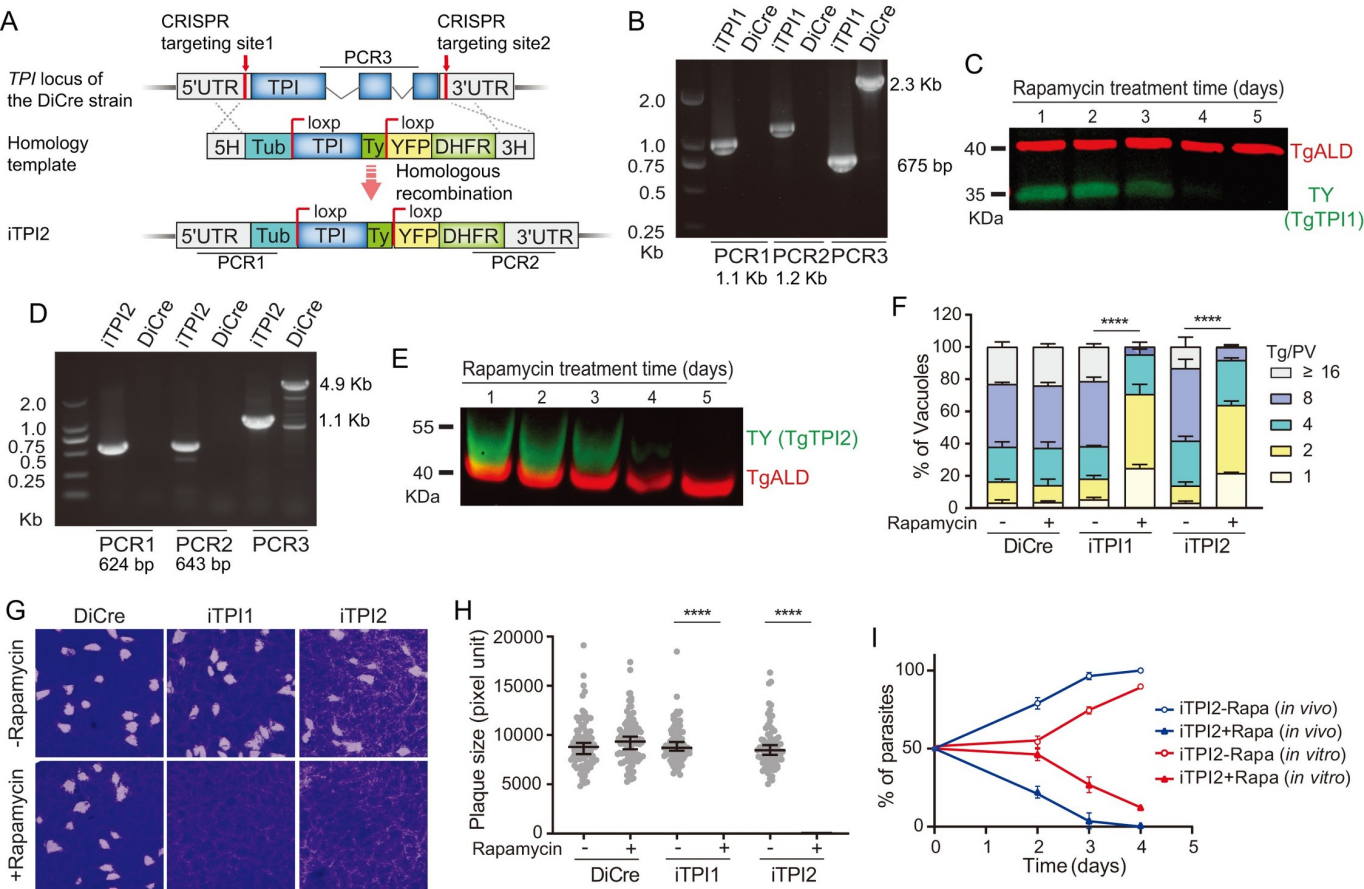
The cytosolic TPI1 and apicoplast TPI2 are both required for *T*. *gondii* growth. A, the DiCre system was used to conditionally knock out the TPI genes. A loxP flanked TPI1 or TPI2 construct was used to replace the corresponding TPI gene using CRISPR/Cas9 assisted homologous recombination in the DiCre-T2A strain. The TPI genes in the resulting iTPI strains could be excised after rapamycin treatment. B-E, diagnostic PCRs (B, D) and Western blotting (C, E) on representative iTPI clones, to demonstrate the correct integration of corresponding constructs and rapamycin dependent depletion of TPI proteins. F, intracellular replication comparing the proliferation rates of indicated strains in the presence and absence of rapamycin. Means **± SEM of three independent experiments, each with three replicates.** ***P < 0.001, two-way ANOVA followed by Tukey’s multiple comparison tests. G, plaque formation of indicated strains on HFF monolayers cultured with or without rapamycin. H, relative sizes (expressed as pixel units) of plaques in G. Median with 95% confidence interval **of more than 100 plaques,** ***P < 0.001, student’s t-test. I. TPI2 is needed for parasite growth both *in vitro* and *in vivo*. The iTPI2 strain was treated with rapamycin for three days to obtain TPI2^-^ parasites (indicated by YFP^+^), which were then mixed with non-treated iTPI2 parasites (which were TPI2^+^ and YFP^-^) at the ratio of 1:1. Then, the mixed culture was either used to infect mice (*in vivo*) or grown in HFF monolayers (*in vitro*) and the ratio between YFP^+^ (TPI2^-^) and YFP^-^ (TPI2^+^) populations was determined by fluorescent microscopy at indicated time point. Means **± SEM of three independent experiments**.

The dispensability of PYK2 and PGK2 promoted us to investigate the physiological roles of other glycolytic isoenzymes in the apicoplast. First, we examined the functions of TPI2 (phenotype score: -4.22). TPI catalyzes the conversion of dihydroxyacetone phosphate (DHAP) to glyceraldehyde 3-phosphate (GA3P). We attempted to knock out *TPI2* directly but were unable to obtain monoclonal gene deletion strains. Therefore, we speculated that TPI2 might play an important role during *T*. *gondii* growth or survival. Alternatively, the DiCre-T2A conditional gene deletion system was used to dissect the role of TPI2 [47]. For comparison, the cytosolic TPI1 (phenotype score: -5.1) was also analyzed by the same genetic manipulation system. The endogenous TPI gene was replaced by a floxed version of the same gene in the DiCre-T2A strain (Fig 4A). Addition of rapamycin would reconstitute a functional Cre recombinase and induce the excision of floxed TPI gene, which was indicated by expression of YFP that was brought to the pTub promoter after TPI excision (S3 Fig). Diagnostic PCRs confirmed the successful floxing of each TPI gene and subsequent Western blotting demonstrated the depletion of TPI expression by rapamycin treatment (Fig 4B–4E). Reduced expression of TPI2 was also confirmed by RT-PCR (reverse transcription-PCR), which demonstrated a sharp decrease of TPI2 mRNA level after rapamycin treatment of the iTPI2 strain (S4 Fig). Typically, gene excision by the DiCre-T2A system is quick and efficient after rapamycin treatment. However, depleting both TPI1 and TPI2 took more than 4 days and the underlying reason is currently unknown. Nonetheless, successful excision of TPI genes allows their functional dissection. After 5 days rapamycin treatment, both the TPI1 and TPI2 conditional mutants (iTPI1 and iTPI2, respectively) displayed a dramatic reduction in intracellular replication, as compared to the corresponding strains without rapamycin treatment (Fig 4F). In addition, TPI1 and TPI2 depletion mutants were completely unable to form plaques in HFF monolayers (Fig 4G and 4H). Together, these results show that both the cytosolic TPI1 and the apicoplast localized TPI2 are required for tachyzoite growth *in vitro*. To check whether TPI2 is also needed for parasite propagation *in vivo*, the iTPI2 strain was treated with rapamycin for three days to obtain TPI2 knockout mutants, which were YFP^+^ (Fig 4A). Then the YFP^+^ mutants were 1:1 mixed with iTPI2 parasites that were not treated with rapamycin, which were YFP^-^ and TPI2^+^. The mixed culture was used to infect mice by intraperitoneal injection. Then, the ratio between YFP^+^ and YFP^-^ parasites extracted from peritoneal fluids was determined at different time points after infection. The results show that the YFP^+^/ TPI2^-^ population decreased rapidly two days after infection and was barely detectable 4 days post infection (Fig 4I). As a control, the same parasite mix was also cultured in vitro to monitor the change between the YFP^+^ and YFP^-^ populations. Again, after 4 days of growth, the culture was dominated by the YFP^-^ population. Therefore, TPI2 is required for tachyzoite proliferation both *in vitro* and *in vivo*.

### Inactivation of the apicoplast localized TPI2 leads to reduced isoprenoid precursor synthesis through the MEP pathway

**Fig 5:**
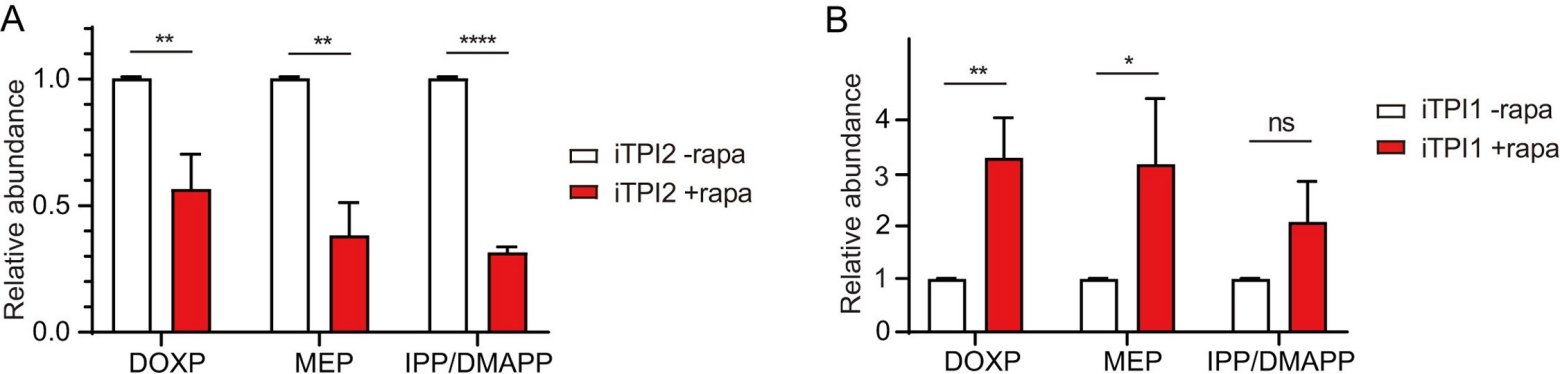
TPI2 depletion reduces isoprenoid precursors synthesis through the MEP pathway. Relative abundance of DOXP, MEP and IPP/DMAPP in the iTPI2 (A) and iTPI1 (B) strains cultured in the presence (+rapa) or absence (-rapa) of rapamycin, as determined by mass spectrometry.

Next, we tried to understand the molecular basis underlying the poor growth of the TPI2 deletion mutants. TPI catalyzes the conversion between DHAP and GA3P [48]. The latter is an important carbon source and one of the initial substrates for the MEP pathway to synthesize isoprenoid precursors. As such, we speculated that TPI2 deletion might lead to reduced GA3P supply in the apicoplast and then decrease activity of the MEP pathway. To test this hypothesis, the relative abundance of four metabolites in the MEP pathway was determined by LC-MS, including 1-deoxy-D-xylulose-5-phosphate (DOXP), MEP, and IPP/DMAPP (which are isomeric to each other). The results showed that significant reduction of DOXP, MEP and IPP/DMAPP levels was observed in the rapamycin treated iTPI2 parasites. The end products of the MEP pathway, IPP/DMAPP, were reduced by almost 70% in the TPI2 depletion mutant, compared to that in the TPI2 expressing parasites (Fig 5A, raw data in S3 Table). For comparison, the abundance of these compounds was also examined in the TPI1 depletion mutant. In contrast to TPI2 depletion, mutants lacking TPI1 had increased levels of MEP pathway intermediates. The levels of MEP and DOXP increased threefold after rapamycin treatment to deplete TPI1 (Fig 5B, raw data in S3 Table), which is likely due to increased DHAP transport into the apicoplast to fuel the MEP pathway when TPI1 was absent in the cytosol to convert DHAP to GA3P. Taken together, these results suggest that TPI2 is indeed critical for isoprenoid precursor synthesis through the MEP pathway, which is essential for parasite growth.

**Fig 6:**
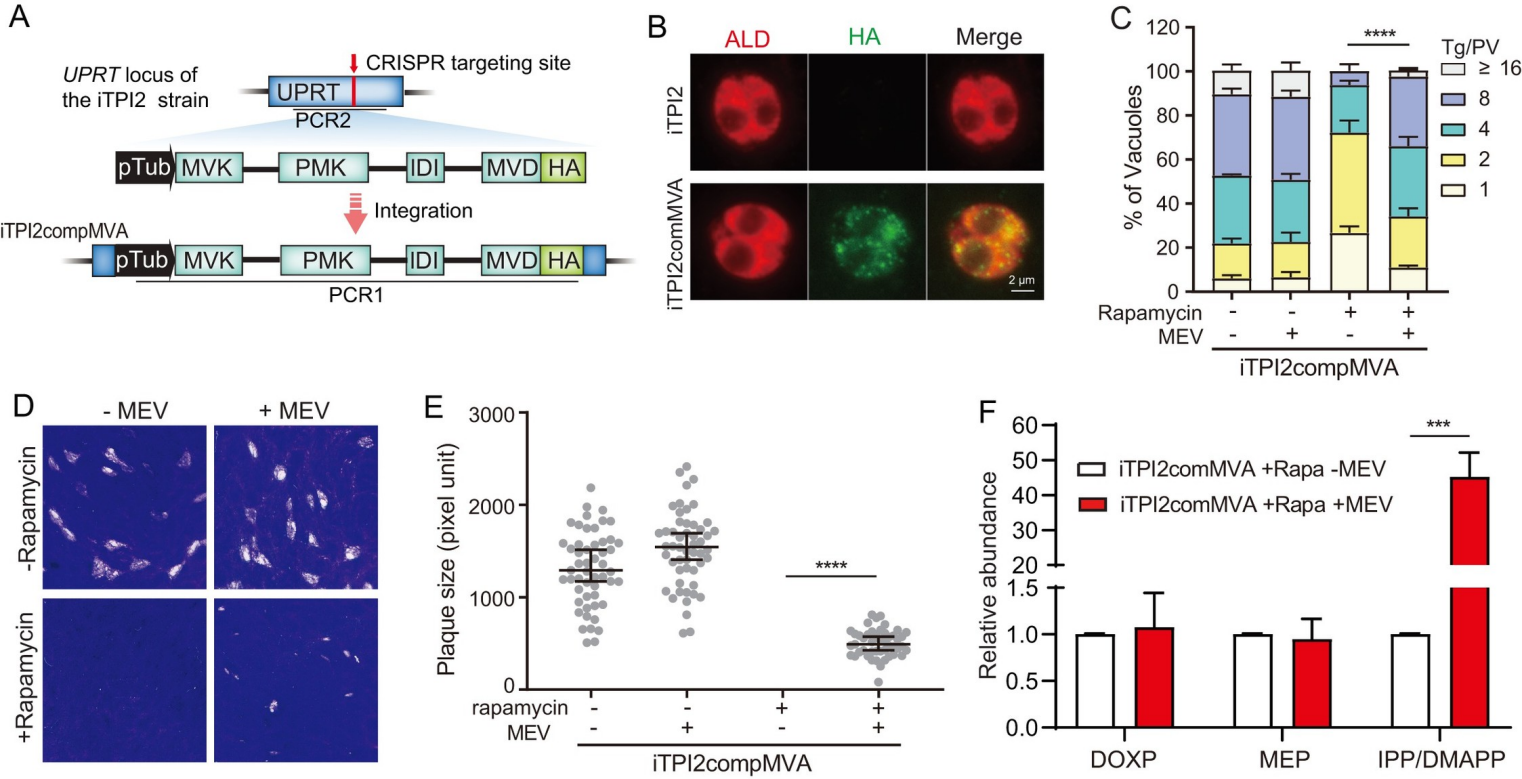
Reconstitution of the MVA pathway significantly improves the growth of TPI2 depletion mutants. A, reconstitution of the MVA pathway into the *UPRT* locus of the iTPI2 strain to generate the iTPI2compMVA line. Following a similar mevalonate bypass system established in *P*. *falciparum*, genes responsible for the last four steps of the MVA pathway (MVK, PMK, MVD, IDI) were expressed from the pTub promoter as a single fusion gene but each enzyme was separated by a short linker. B, IFA demonstrating the successful reconstitution of the MVA pathway in the cytosol of iTPI2compMVA, which was determined by probing the expression of HA that was fused to the last enzyme in the polycistronic construct. ALD was used as a cytosolic marker. C, intracellular replication rates of the iTPI2compMVA strain cultured under indicated conditions. Means **± SEM of three independent experiments, each with three replicates.** ***P < 0.001, two-way ANOVA followed by Tukey’s multiple comparison tests. MEV, mevalonate. D, overall growth of the iTPI2compMVA strain grown under indicated conditions as determined by plaque assays. E, relative size (pixel units) of plaques derived from D. F, relative abundance of DOXP, MEP and IPP/DMAPP in iTPI2compMVA parasites treated with rapamycin and cultured in the presence or absence of MEV. The level of each metabolite in the absence of MEV was set as 1 and used to normalize that in the presence of MEV. Means **± SD of three independent experiments,** ***P < 0.001, student’s t-test.

To further confirm whether reduced synthesis of isoprenoid precursors IPP and DMAPP were responsible for the growth defects of the TPI2 depletion mutant, we sought to determine whether supply of IPP and DMAPP by other means would rescue its growth. While *Plasmodium* parasites take up IPP efficiently from the medium and IPP supplementation can rescue the growth of parasites devoid of the apicoplast organelle, *T*. *gondii* parasites do not seem to import IPP effectively [49,50]. As such, adding IPP to culture medium would not be able to rescue the growth of the TPI2 depletion mutant. Alternatively, we reconstituted part of the mevalonate (MVA) pathway in the cytosol of the iTPI2 strain, following a similar design in the mevalonate dependent apicoplast bypass line in *P*. *falciparum* [39]. The MVA pathway uses totally different enzymes than the MEP pathway to synthesize isoprenoid precursors IPP and DMAPP, using acetyl-CoA as substrates. In our design, the last four steps of the MVA pathway that convert mevalonate to IPP/DMAPP was reconstituted in the iTPI2 strain. The four enzymes (mevalonate kinase (MVK), phosphomevalonate kinase (PMK), mevalonate 5-diphosphate decarboxylase (MVD) and isopentenyl diphosphate isomerase (IDI)) were expressed as a single fusion gene transcribed from the pTub promoter, each was separated by a flexible linker (Fig 6A). Expression of the reconstituted MVA in the iTPI2compMVA strain was confirmed by immunofluorescent staining, which showed expression of the HA tag that was fused to the last enzyme in recombinant construct (Fig 6B). Without rapamycin to deplete TPI2, mevalonate had little effect on the replication rates of the iTPI2compMVA strain. However, in the presence of rapamycin, mevalonate supplementation greatly improved parasite replication (Fig 6C). Similarly, the plaque formation defects caused by TPI2 depletion was significantly restored by mevalonate supplementation, although not to the level of full rescue (Fig 6D and 6E). Metabolic measurements also show that mevalonate supplementation increased the cellular IPP/DMMAPP level by more than 40-fold in the TPI2 depletion mutants. But it did not affect the abundance of DOXP or MEP (Fig 6F, raw data in S3 Table). These results suggest that reduced IPP/DMMAPP production is a major cause of the growth defects of TPI2 depleted mutants. Nonetheless, the lack of full rescue of iTPI2compMVA by mevalonate implies that TPI2 may be additional roles that are independent of the MEP pathway, which deserves further investigation.

We also examined the impact of TPI2 depletion on *de novo* fatty acids in the parasites. To do this, the iTPI2 strain pretreated with rapamycin was cultured in medium containing 8 mM ^13^C_6_-glucose for 2 days. Then, fatty acids were extracted and the incorporation of ^13^C into different fatty acid species was determined by LC-MS. The results show that, compared with the parasite that did not receive rapamycin treatment, rapamycin treated parasites had very similar distribution of ^13^C labeled fatty acids, including C14:0 and C16:0 that were mainly synthesized in the apicoplast (S5 Fig). These results suggest that TPI2 depletion does not affect fatty acid synthesis in the parasites.

### GAPDH2 is also required for apicoplast metabolism and parasite growth

**Fig 7:**
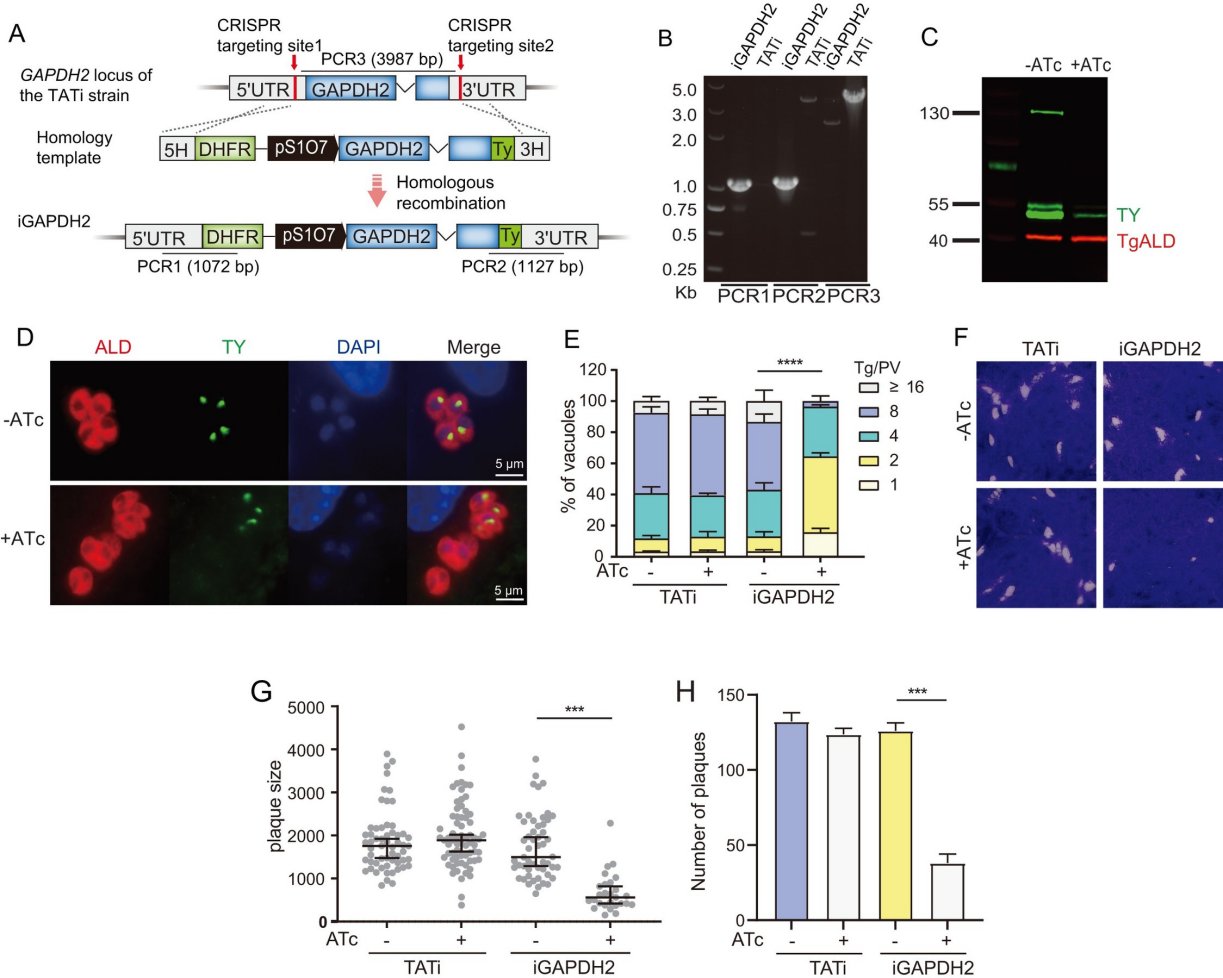
Depletion of GAPDH2 leads to impaired parasite growth. A, strategy used to construct the iGAPDH2 strain, which contained an anhydrotetracycline (ATc) regulatable promoter pS1O7 to regulate the expression of *GAPDH2*. The endogenous *GAPDH2* gene was replaced with a construct that contained Ty tagged *GAPDH2* expressed from pS1O7 and the selection marker *DHFR*. B, diagnostic PCR on an iGAPDH2 clone. C, Western blotting demonstrating the reduction of *GAPDH2* expression after ATc treatment, as probed by a Ty antibody. D, IFA checking the expression of *GAPDH2* in the presence or absence of ATc. Note that after five days ATc treatment, about 40% of parasites still expressed *GAPDH2* (as determined by visible Ty staining signal in IFA), although the expression level might not be as high as that in parasites without ATc treatment. E, intracellular replication assay comparing the proliferation rates of iGAPDH2 in the presence or absence of ATc treatment. ***P < 0.001, two-way ANOVA followed by Tukey’s multiple comparison tests. F, plaque assay estimating the overall growth of indicated strains grown with or without ATc. G-H, relative sizes (G) and numbers (H) of plaques derived from F. Median with 95% confidence interval **of more than 100 plaques (G),** Means **± SD of three replicates (H),** ***P < 0.001, student’s t-test.

GAPDH catalyzes the conversion of GA3P to 1,3-bisphosphoglycerate (1,3-PG), with the generation of reducing power in the form of NADH or NADPH. *T*. *gondii* encodes two GAPDHs and sequence analyses on the key residues that determine NAD^+^ or NADP^+^ cofactor selectivity suggested that GAPDH1 was probably NAD^+^ specific and GAPDH2 could use both but likely preferred NADP^+^ [51]. GAPDH1 has been shown to be essential for tachyzoite growth in *T*. *gondii* [52]. To investigate the role of GAPDH2 (phenotype score: -4.4), it was subjected to gene disruption studies. Direct deletion of GAPDH2 by CRISPR/Cas9 mediated homologous gene replacement was not successful, suggesting that it might have critical roles for parasite growth. Then, we used conditional gene regulation approaches to deplete its expression. Both the DiCre-T2A [47] and the auxin inducible degradation (AID) systems [53] failed to regulate GAPDH2 expression. In the former case, rapamycin treatment did not induce excision of floxed GAPDH2. Whereas in the latter, auxin treatment had little effect on the protein level of GAPDH2. Nonetheless, we managed to regulate GAPDH2 expression through the TATi system that used an ATc regulatable promoter pS1O7 to control its transcription (Fig 7A). Diagnostic PCRs confirmed the replacement of the GAPDH2 locus with a pS1O7 regulated GAPDH2 construct in the iGAPDH2 strain (Fig 7B). Western blotting also showed reduced expression of GAPDH2 after ATc treatment. Nonetheless, even with 5 days ATc treatment, residual amount of GAPDH2 was still detectable (about 10% of that without ATc treatment) (Fig 7C). This is largely caused by the fact that ATc treatment failed to down regulate GAPDH2 expression in nearly 40% of the iGAPDH2 parasite population, because GAPDH2 could still be detected in these parasites by IFA (Fig 7D). These results are consistent with the RT-PCR data, which showed roughly 50% reduction of GAPDH2 mRNA after ATc treatment (S4 Fig). We have subcloned the iGAPDH2 strain multiple times to obtain clonal lines, but each time there were certain fraction of the parasites that still contained visible levels of GAPDH2 after ATc treatment. The underlying reason for such incomplete regulation is currently unknown.

Although not perfect, the iGAPDH2 strain offered an opportunity to assess the physiological role of GAPDH2 during parasite growth. First, an intracellular replication assay was used to estimate the role of GAPDH2 during parasite proliferation. The results showed that ATc treatment significantly reduced the replication rates of iGAPDH2 parasites (Fig 7E). It is worth noting that in this experiment, only the parasites that did not express detectable GAPDH2 (the ones lacking detectable Ty staining signal when the parasites were stained with anti-ALD and anti-Ty antibodies) were included for analysis for the “iGAPDH2 + ATc” group, given the uneven depletion issue described above. Similarly, plaque assays indicated that GAPDH2 suppression significantly reduced plaquing efficiency of the parasites, producing less and smaller plaques (Fig 7F–7H). These results demonstrate that GAPDH2 is critical for the growth of *T*. *gondii* tachyzoites *in vitro*.

**Fig 8:**
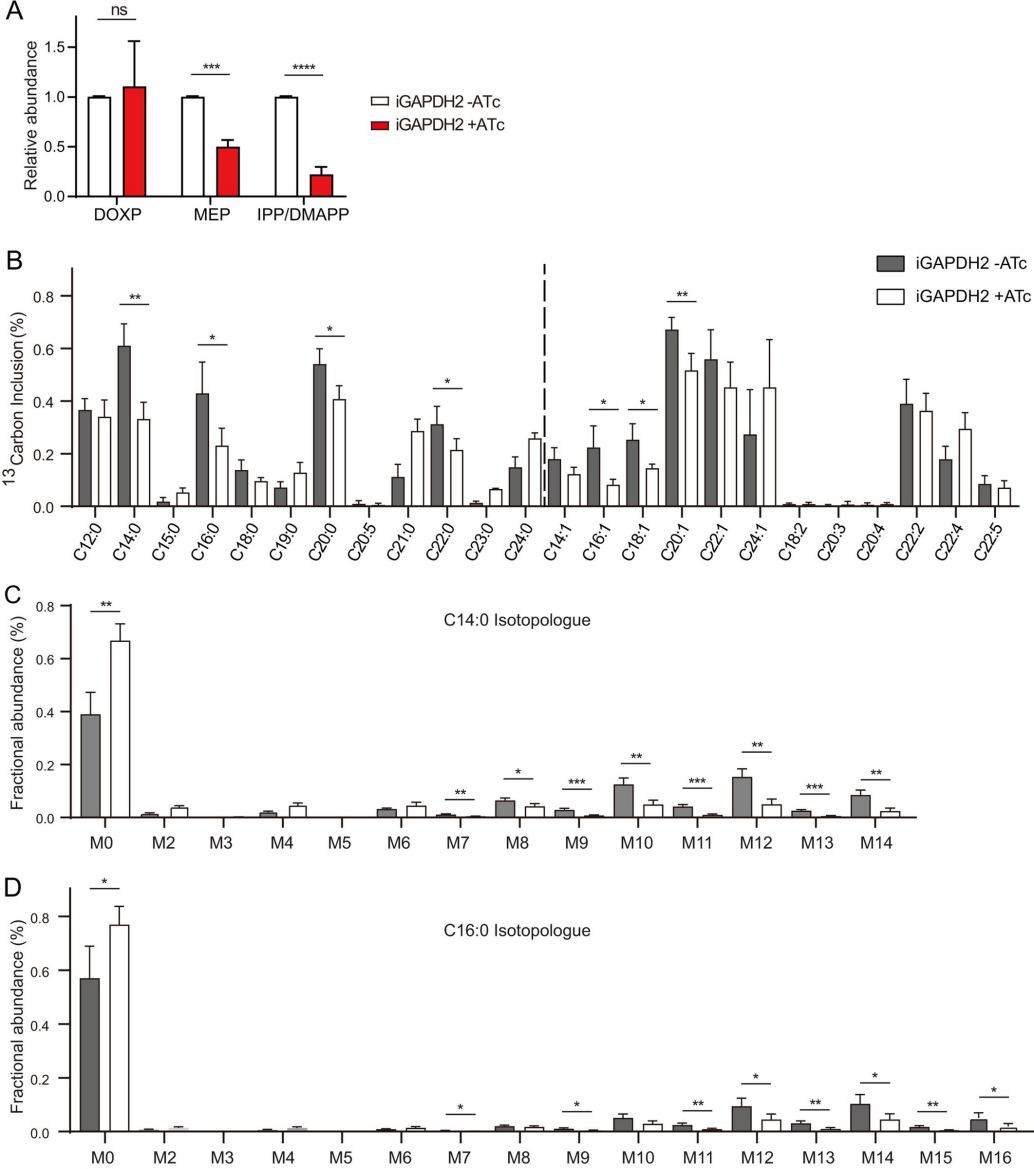
GAPDH2 is needed for the synthesis of isoprenoid precursors and fatty acids. A, relative levels of DOXP, MEP and IPP/DMAPP in iGAPDH2 parasites treated with or without ATc, as determined by mass spectrometry. Means **± SD of three independent experiments,** ***P < 0.001, student’s t-test. B, reduced fatty acids synthesis in the GAPDH2 depletion mutants. The iGAPDH2 parasites were pretreated with or without ATc for 3 days. Then the parasites were cultured under the same pretreatment conditions for another two days in medium containing 8 mM ^13^C_6_-glucose. Subsequently, fatty acids were extracted and analyzed by LC/MS. For each fatty acid species, the ^13^C labeled fraction (as % of the total) was plotted. C-D, ^13^C inclusion in each isotopologue of myristic acid (C14:0) and palmitic acid (C16:0). M0 –M16 denote the number of carbon atoms that were ^13^C labeled. Means **± SD of three independent experiments,** *P < 0.05, **P < 0.01, ***P < 0.001, student’s t-test.

To further understand the molecular basis underlying the reduced fitness of the GAPDH2 depletion mutants, we examined its impact on apicoplast metabolism. Since GAPDH2 is likely to be involved in reducing power production in the apicoplast, we examined two pathways that need reducing power, the MEP pathway, and the FASII pathway. ATc treatment to suppress GAPDH2 reduced MEP level by 50% and the IPP/DMAPP level by almost 80%. But it did not affect the abundance of DOXP (Fig 8A, raw data in S3 Table). These results are consistent with the role of GAPDH2 in reducing power production, since DOXP production is the first step of the MEP pathway and it does not require reducing power, whereas the second step that produces MEP, as well as the last two steps that generate IPP/DMAPP need NADPH as reducing power. To assess the role of GAPDH2 in FASII, ^13^C_6_-glucose was used to label intracellular iGAPDH2 parasites that were treated with or without ATc. Then, the incorporation of ^13^C into fatty acids was determined by LC-MS. In contrast to TPI2 depletion, GAPDH2 suppression reduced the incorporation of ^13^C into diverse fatty acid species (Fig 8B), including saturated and unsaturated fatty acids. Specifically, GAPDH2 suppression led to roughly 40% reduction of ^13^C incorporation into C14:0 and C16:10, two main products of the FASII pathway in the apicoplast. A detailed look at the isotopologue distribution in C14:0 and C16:10 further confirmed the reduced ^13^C incorporation into these two fatty acid species, particularly the isotopologues with 10 or more ^13^C atoms. Taken together, these data demonstrate that GAPDH2 is needed for optimal activities of the MEP and the FASII pathways in *T*. *gondii* apicoplast, likely by providing reducing power in the form of NADPH.

## Discussion

**Fig 9:**
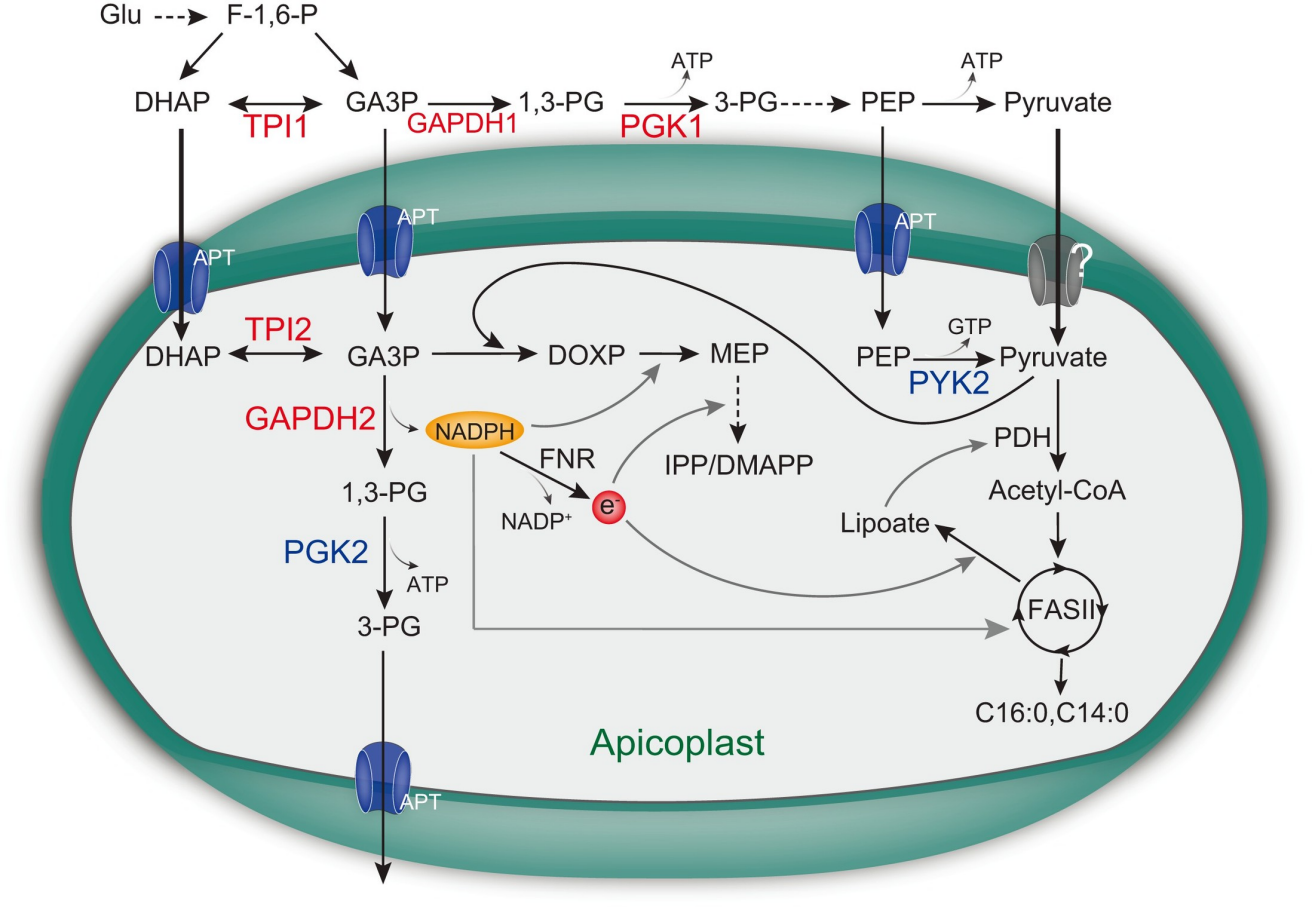
A model for the functions of glycolytic enzymes in *T*. *gondii* apicoplast. The apicoplast phosphate translocator (APT) imports a number of glycolytic intermediates into the apicoplast, including DHAP, GA3P and PEP. TPI2 catalyzes the interconversion of DHAP and GA3P, providing carbon source (GA3P) for the MEP pathway. The other substrate for the MEP pathway, pyruvate, may be acquired by multiple routes, through the conversion of PEP catalyzed by PYK2 and the direct import from cytosol by an unknown transporter. Dispensability of PYK2 suggests that direct import is probably the main source for pyruvate in the apicoplast. In addition, the lack of a strong growth defect of the *Δpyk2-Δpgk2* mutant indicates additional source of energy to power apicoplast metabolism. GAPDH2 catalyzes the conversion of GA3P to 1,3-PG, along with the generation of reducing power in the form of NADPH, which is needed in the MEP pathway (by DOXPRI) and the FASII pathway (by FabG). In addition, NADPH may be a critical donor for electrons, via ferredoxin-NADP^+^ reductase (FNR), to electron requiring enzymes like lipA during lipoate synthesis and IspG in the MEP pathway. The “?” mark denotes an unknown transporter that imports pyruvate from the cytosol.

Apicoplast is an essential organelle with key metabolic functions in most apicomplexan parasites [12,36,54,55]. But how exactly the metabolic network in functioning and the physiological significances of each enzyme or pathway are not well understood. In this study, confirmed the localization of four glycolytic isoenzymes in the apicoplast of *T*. *gondii*. Subsequent genetic and biochemical studies revealed that TPI2 and GAPDH2 were essential for *T*. *gondii* growth, whereas PYK2 and PGK2 were dispensable. Taken together, our results point to a refined model for metabolism in *T*. *gondii* apicoplast (Fig 9). The glycolytic intermediates DHAP and GA3P generated in the cytosol can both be imported to the apicoplast by the apicoplast phosphate translocator (APT) [41]. Because cytosolic GA3P may be quickly used by glycolysis in the cytosol, DHAP may be the main triose-phosphate imported to the apicoplast. From there, the apicoplast localized TPI2 converts DHAP to GA3P, which serves as a key carbon source for the MEP pathway [22]. As a result, TPI2 depletion led to reduced MEP activity and decreased levels of IPP/DMAPP. Reconstitution of the MVA pathway in the TPI2 depletion mutants restored IPP/DMAPP production and significantly rescued its growth defects. In addition to being used as a carbon source for the MEP pathway, GA3P is also used by GAPDH2 in the apicoplast to produce 1,3-PG and NADPH. The NADPH generated by GAPDH2 is predicted to be a key source of reducing power for a number of metabolic reactions in the apicoplast [56]. Meanwhile, NADPH can also donate electrons, via the ferredoxin-NADP^+^ reductase (FNR)—ferredoxin redox system, to electron requiring enzymes [57]. In *T*. *gondii* apicoplast, the LipA protein involved in lipoate synthesis and the IspG and IspH involved in the MEP pathway, all require electrons for activity [56]. As such, GAPDH2 depletion resulted in reduced NADPH generation in the apicoplast, leading to decreased MEP and FASII activity. Our updated model also predicts the presence of a pyruvate transporter in apicoplast membranes, because PYK2 is the only known reaction that generates the essential metabolite pyruvate in the apicoplast but its deletion did not have a noticeable impact on parasite growth [12]. In addition, the tolerance to simultaneous deletion of PYK2 and PGK2 suggests that there are probably other pathways for energy supply in *T*. *gondii* apicoplast, which deserve further investigation.

Although the apicoplast has important metabolic functions in both *T*. *gondii* and *Plasmodium* parasites, fundamental differences also exist in apicoplast metabolism between these two parasites. Related to the work in this study, *Plasmodium* parasites only have two glycolytic enzymes localized to the apicoplast, namely TPI and PYK2 [17,18]. Nonetheless, just opposite to the cases in *T*. *gondii*, TPI seems to be dispensable in blood stage *P*. *falciparum*, whereas PYK2 is essential. Using the apicoplast bypass line PfMev that expresses the four enzymes from the MAV pathway that convert mevalonate into isoprenoid precursors (similar to our iTPI1compMVA strain) [39], TPI and PYK2 were successfully deleted, respectively. Later it was found that the growth of PfMev/*Δpyk2* was dependent on mevalonate supplementation, but that of PfMev/*Δtpi* was not, under conditions that induced apicoplast loss. These data suggest that the apicoplast localized TPI is neither required for apicoplast maintenance nor for the MEP pathway in *P*. *falciparum* [39]. PYK2, on the other hand, is able to convert all NDPs and dNDPs to corresponding NTPs and dNTPs without strong specificity [19]. As such, it provides these nucleotide triphosphates for the apicoplast to replicate and transcribe its genome, as well as to support other metabolic activities. Therefore, it is essential for apicoplast maintenance and parasite survival. In *T*. *gondii*, TPI2 is essential but PYK2 is not. *T*. *gondii* PYK2 seems to have a strong preference for GDP over ADP as the substrate [45]. Nonetheless, dispensability of PYK2 suggests that whatever nucleotides it generates, there are probably additional ways to provide them. In terms of the source of NTPs and dNTPs for apicoplast genome replication and transcription in *T*. *gondii*. There is no apparent source yet based on the currently known proteins in the apicoplast organelle. It is possible that they are imported into the apicoplast by transporter(s), as nucleotide transporters are widely present in chloroplasts [58]. Further investigations are needed to check this possibility.

While TPI2 is needed for optimal activity of the MEP pathway, likely by providing the key substrate GA3P, it is not required for the FASII pathway. TPI2 depletion did not affect the fatty acid synthesis activities in the parasites. This is somewhat surprising, particularly when inactivation of the downstream enzyme GAPDH2 did reduce FASII activity. Since GA3P is also the substrate for GAPDH2, reduced GA3P supply after TPI2 depletion would have also caused reduced flux through GAPDH2, leading to decreased supply of reducing power and reduced MEP/FASII activity. There are a couple of possibilities to explain this observation. First, GAPDH2 may have a higher affinity for GA3P than DOXP synthase, which catalyzes the first step in the MEP pathway. In the absence of TPI2, limited amount of GA3P can be imported into the apicoplast by APT. Higher affinity of GAPDH2 for GA3P makes the supply of GA3P for the GAPDH2 pathway less affected than for the MEP pathway. As such, depletion of TPI2 only reduced the activity of the MEP pathway but not the FASII. The second possibility is that the production of GA3P may not be the only important role of TPI2 in the apicoplast. One observation that supports this is that although MVA reconstitution drastically increased the abundance of IPP/DMAPP, it did not fully rescue the growth defects of the TPI2 depletion mutant. Although there may be other possibilities, like the ratio between IPP and DMAPP generated by the reconstituted MVA pathway did not satisfy the needs of *T*. *gondii*, the lack of full rescue certainly points to a possibility that reduced MEP activity may not be the sole defect of the TPI2 depletion mutant. The additional roles of TPI2, if any, needs further investigation.

The source of reducing power in the apicoplast is long debating. It is clear that many metabolic pathways, including MEP, FASII, and the ferredoxin redox system [22,59,60], all need reducing power for proper function. Yet the source has not been fully defined in any of the apicomplexan parasites. Reducing power in the forms of both NADH and NADPH is needed in the apicoplast. NADH can be produced by the apicoplast localized pyruvate dehydrogenase complex (PDH). Yet, PDH is dispensable in tachyzoites and blood stage *Plasmodium spp* [36,61]. Perhaps FASII is the main metabolic pathway that needs NADH, so that the growth defects of the *T*. *gondii* mutants lacking PDH could be largely rescued by the supply of exogenous fatty acids. Alternatively, there may be other sources of apicoplast NADH like import from the cytosol. The source of NADPH is more puzzling. We show here that GAPDH2 is probably a major source for NADPH in *T*. *gondii* apicoplast. Canonical cytosolic GAPDH enzymes use NAD^+^ as a cofactor to produce NADH. Chroloplast GAPDH enzymes can use both NAD^+^ and NADP^+^ [51,62]. Sequence analyses and biochemical studies have revealed some of the key residues that determine the substrate specificity. According to those findings, *T*. *gondii* GAPDH2 is highly likely to use both NAD^+^ and NADP^+^, thus being able to generate NADPH [51,62]. Nonetheless, experimental evidence to confirm its NADPH generating activity is needed in the future. In addition to GAPDH2, an apicoplast localized NADP^+^-dependent isocitrate dehydrogenase (IDH) (TGME49_266760) may also provide NADPH for *T*. *gondii* apicoplast [63]. The physiological significance of this IDH has not been examined, but the phenotype score (0.35) calculated from a genome-wide genetic screen suggests that it is likely dispensable. Interestingly, *P*. *falciparum* does not have apicoplast localized GAPDH or IDH enzymes, although mutations in the cytosolic GAPDH were recently shown to confer resistance to fosmidomycin, a potent inhibitor of the MEP pathway and antimalaria drug. Those GAPDH mutants were still catalytically active but had altered enzymatic cooperativity that could relieve the metabolic imbalance caused by fosmidomycin, likely by changing the substrate availability and increasing glycolytic substrates to the MEP pathway [64]. In seeking of NADPH in *Plasmodium* spp., an NAD(P)^+^ transhydrogenase (NTH) that can catalyze the interconversion between NADPH and NADH was recently described in *P*. *berghei*. It was localized to the crystalloids of oocysts and the apicoplast of sporozoites. Genetic studies showed that NTH was required for optimal sporozoite development and transmission to vertebrate hosts. Nonetheless, the *Δnth* mutants had normal asexual growth at the blood stage and gametocyte development in mice [56,65]. As such, how NADPH is provided in blood stage parasites is still an open question and import from the cytosol is another possibility.

## Materials and methods

### Ethics statement

All mice used in this study were purchased from the Center of Disease Control and Prevention in Hubei Province and maintained under standard conditions according to the regulations specified by Administration of Affairs Concerning Experimental Animals. All animal experiments were approved by the ethical committee of Huazhong Agricultural University (permit no. HZAUMO-2022-0090).

### Parasites strains and experimental animals

The RH *Δhxgprt* (RH), TATi [66], DiCre-T2A [47] and RH *Δku80* strains were used to construct genetically modified strains. All parasite strains were maintained in human foreskin fibroblast (HFF) cells (purchased from ATCC, USA), which were cultured in DMEM medium (Life Technologies, USA) containing 2% fetal bovine serum and 1% penicillin−streptomycin (Life Technologies, USA). Anhydrotetracycline (ATc) (Takara Bio USA, Inc., USA) at final concentration of 1 μg/mL was used to suppress the expression of pS1O7 regulated genes. Final concentration of 50 nM rapamycin (Aladdin, China) was used to induce excision of target genes engineered in the DiCre-T2A system. 10 mM mevalonate was used to induce IPP/DMAPP synthesis via the MVA pathway in the iTPI2compMVA strain. Seven-week-old female ICR mice were used for virulence tests of *T*. *gondii* strains. Nine-week-old Kunming mice were used to produce polyclonal antibodies against PGK2.

### Construction of plasmids

All plasmids used in this study and brief description of their construction methods are listed in S1 Table. All primers used are listed in S2 Table. Site specific CRISPR plasmids were constructed by replacing the *UPRT* targeting guide RNA (gRNA) in pSAG1-Cas9-sgUPRT with corresponding gRNAs by site-directed mutagenesis, as described previously [67]. In cases (for the construction of iTPI1, iTPI2 and iGAPDH2) where dual gRNA in one CRISPR plasmid was used, two single gRNA CRISPR plasmids were constructed first. Then, the pU6-gRNA2 fragment from pSAG1-Cas9-U6: gRNA2-sgTPI was amplified and cloned into KpnI and XhoI digested pSAG1-Cas9-U6: gRNA1-sgTPI. All homology template plasmids used for targeted gene editing were constructed by multi-fragment ligation using the ClonExpress One Step Cloning Kit (Vazyme Biotech, Co. Ltd., China). Detailed information on what fragments each plasmid contained and how they were obtained is provided in S1 Table. To construct the plasmid for MVA pathway reconstitution, the codon optimized synthetic sequences coding the four genes of the MVA pathway was cloned into the vector pG265-pTub-UPRT that delivering the MVA genes to the *UPRT* locus of the parasites. The plasmid pET-28a-PGK2-truncation which expressed a PGK2 derived polypeptide was constructed by cloning the corresponding PGK2 fragment (from AA 100 to 307) amplified from RH cDNA into the pET-28a vector, through a one-step cloning kit (Vazyme Biotech, China). The plasmids pET-28a-2HIS-SUMO-PGK1 and pET-28a-2HIS-SUMO-PGK2 were constructed in similar ways, by cloning the coding sequences of PGK1 or PGK2 amplified from cDNA of RH into pET-28a-2HIS-SUMO respectively.

### Construction of genetically modified *T*. *gondii* strains

To tag endogenous glycolytic genes with smHA or Ty, each tagging construct (containing short homologous arms for the target locus, the epitope tag and *DHFR* selection cassette) was amplified from pPUC19-Ty-3’UTR-DHFR [68] or pSL24m-Linker-smFP-DHFR-LoxP-T7 (a gift from Dr. Shaojun Long, China Agricultural University) (primers listed in S2 Table) and co-transfected into RH *Δku80* tachyzoites with the corresponding CRISPR plasmid. Subsequently, transfectants were selected with 1 μM pyrimethamine for 10 days and then examined by immunofluorescent assays (IFA).

To construct the PGK2 knockout line *Δpgk2* and the double deletion strain *Δpgk2-Δpyk2*, the DHFR and CAT containing homologous gene replacement constructs were electroporated into RH and *Δpyk2* strains respectively, along with the PGK2 targeting CRISPR plasmid. Subsequently the cultures were selected with 1 μM pyrimethamine or 30 μM chloramphenicol, single cloned by limiting dilution and examined by diagnostic PCRs to identify the correct clonal mutants.

The iGAPDH2 strain was constructed by deleting the entire endogenous GAPDH2 locus and replacing it with a Ty tagged GAPDH2 driven by an ATc regulatable promoter pS1O7, along with the pyrimethamine resistant marker *DHFR* (Fig 7A). The homology template and the double gRNA containing CRISPR plasmid were electroporated into purified tachyzoites of the TATi line and selected with 1 μM pyrimethamine. Single clones were obtained and screened as above. The iPGK1 line was constructed in a similar way, but a single gRNA containing CRISPR plasmid targeting the 5’ end of PGK1 was used. The iTPI1 and iTPI2 strains were constructed by co-transfecting the corresponding floxing constructs with the CRISPR plasmids (each contained two gRNAs) into the DiCre-T2A strain and selected 1 μM pyrimethamine. The detailed design was illustrated in Fig 4A. The iTPI2compMVA strain was constructed by inserting the fragment expressing four MVA genes (MVK, PMK, MVD, IDI) into the *UPRT* locus of the iTPI2 strain. The MVA gene containing fragment was transfected into iTPI2 tachyzoites along with a *UPRT* targeting CRISPR plasmid. Transfectants were selected with 10 μM 5-fluorodeoxyuridine and single clones obtained were examined by diagnostic PCR and IFA before further use.

### Immunofluorescent assays (IFAs)

IFAs were performed following previously described protocols [12]. The primary antibodies used in this research are as follows: mouse anti-Ty monoclonal antibody (provided by Dr. David Sibley at Washington University School of Medicine), HA monoclonal antibody (Medical & biological laboratories Co., Ltd, Japan), rabbit anti-ALD (provided by Dr. David Sibley at Washington University School of Medicine), rabbit anti-CPN60 (provided by Dr. Honglin Jia at Harbin Veterinary Research Institute in China), mouse anti-HSP60, mouse anti-PGK2 (generated in this study using AA 100–307 of recombinant *T*. *gondii* PGK2 as antigen) and mouse anti-PYK2 [69]. Alexa-594 and -488 conjugated secondary antibodies (Life Technologies, USA) were used to detect primary antibodies. Fluorescent images were acquired by the BX53 fluorescence microscope (Olympus life Science, Japan) equipped with an Axiocam 503 monochrome camera (Carl Zeiss Inc., Germany), and slightly adjusted in the ZEN software (Carl Zeiss Inc., Germany) for optimal contrast and brightness. To examine the colocalization of smHA or Ty tagged glycolytic enzymes with cytosol (ALD) ([10]), mitochondrion (HSP60) ([70]) or apicoplast (CPN60) ([71]) specific markers, fluorescent images were acquired with auto-exposure time and analyzed by Pearson’s correlation tests to assess the degree of colocalization. Briefly, the images merged by ZEN were imported into the Fiji software and the region containing the PV was selected. Then, the signals from the green and the red channels in selected area were subjected to Pearson’s correlation analysis using the colocalization finder plugin and the Pearson correlation coefficients (PCCs) were calculated.

### Western blotting

Freshly egressed parasites (1×10^7^ per sample) were purified by filtration to remove host cell debris, resuspended in 40 μl RIPA lysis buffer (Beyotime, China) and sonicated for 30 min in a water bath sonicator. Then, 10 μl DTT and 50 μl 2×SDS buffer were added to each sample and boiled for 10 min. Protein samples (10 μl each) were separated on SDS-PAGE gels and transferred to nitrocellulose membranes. Subsequently, the membranes were probed with indicated primary antibodies. Two different strategies were used to detect primary antibodies. For chemiluminescent detection, HRP-conjugated sheep anti-rabbit or sheep anti-mouse IgG (Epizyme, China) secondary antibodies (at the dilution of 1:2000) were used and the blots were developed by a BeyoECL Moon kit (Beyotime, China). For fluorescent detection, IRDye 800CW sheep anti-mouse IgG and IRDye 680RD sheep anti-rabbit IgG (LI-COR bioscience, USA) secondary antibodies (at the dilution of 1:5000) were used. The blots were scanned by a Tanon 5200 chemiluminescence imager (Tanon, China) and a Typhoon5 laser-scanner (GE Healthcare, USA).

### Protein purification and enzymatic assays

The plasmids that allowed the expression of PGK1 (pET-28a-2HIS-SUMO-PGK1) and PGK2 (pET-28a-2HIS-SUMO-PGK2) were individually transformed into BL21(DE3) competent cells, and the expression of recombinant proteins was induced with 1 mM IPTG at 37°C. Subsequently, bacterial cells were lysed in a French press and recombinant proteins were purified using the BeyoGold His-tag Purification Resin (Beyotime, China), following the instructions from the manufacturer. The concentrations of purified protein were determined by SDS-PAGE with different concentrations of bovine serum albumin as standards. Then, the enzymatic activity of purified proteins was tested using a colorimetric PGK activity assay kit (Bioisco, China). The reactions were performed in 100 μl assay buffer containing 5 μg/ml protein (the enzyme concentration was optimized by trial experiments to allow linear measurements within the first 2 minutes of the reactions) and different concentrations of 3-GP (0, 60, 120, 180, 240, 360, 480, 600, 720, 840 or 960 μM). The OD_340_ of the reaction samples was monitored for 2 min and the slopes of the OD_340_ curves were used to calculate the PGK activity using the following equation: PGK activity (nmol/min/mg protein) = ΔOD_340_÷(ε×d) ×V_total_ ÷ (Cpr × Vol sample) ÷T, ε = 6.22×10^3^ L/mol/cm, d = 0.2 cm, Cpr: 5 ng/ml, V_total_ = 100 μl, Vol sample = 10 μl, T = 2 min (within this time period, the slopes of the OD_340_ curves remained constant). Each enzyme was tested three times independently.

### Parasite growth assays

Plaque assays that estimate the overall growth of parasites *in vitro* under indicated conditions were performed as previously described [12]. Intracellular replication assays that assess the proliferation rates of parasites were also done following previously established protocols [72]. For the iTPI1, iTPI2 and iGAPDH2 strains, parasites were pretreated with rapamycin (iTPI1 and iTPI2) or ATc (iGAPDH2) for five days before assessed by intracellular replication assays. To examine the replication of the GAPDH2 depleted parasites, the iGAPDH2 strain was pretreated with or without ATc for 5 days and then used to infect HFF monolayers. Invaded parasites were allowed to replicate for 24 hours under the corresponding pretreatment conditions. Then the samples were fixed and stained with both anti-TgALD and anti-Ty antibodies. To determine the replication rate of the ATc treated parasites (the +ATc group), only parasites that were TgALD^+^ and Ty^-^ were included for analysis. For both plaque and replication assays, each strain and condition were repeated at least three times independently, each with three replicates.

To examine the proliferation of TPI2 depleted parasites in mice, the DiCre-TPI2 strains were first treated with rapamycin for 3 days to obtain YFP^+^ (TPI2^-^) parasites. Then the YFP^+^ parasites were mixed with DiCre-TPI2 parasites that did not receive rapamycin treatment (YFP^-^ TPI2^+^) at 1:1 ratio and used to infect ICR mice through intraperitoneal injection (10^5^ parasites per mouse, 12 mice were used). Then, the percentages of YFP^+^ and YFP^-^ populations in parasites collected from mouse intraperitoneal fluids were determined by microscopy 2-, 3-, and 4-days post infection. The mixed parasites (10^5^ parasites at 1:1 ratio) were also used to infect HFF cells *in vitro* to serve as a parallel reference and changes in the YFP^+^ and YFP^-^ populations were monitored similarly.

### Virulence tests in mice

Freshly egressed parasites were purified and counted, and then injected into 7-week-old female ICR mice (100 parasites per mouse and 10 mice per strain) intraperitoneally. Subsequently, the symptoms of infection and survival of infected mice were monitored daily. Mice were euthanized once signs suggestive of irreversible death were observed. Cumulative mortality was graphed as Kaplan-Meier survival plots and analyzed in Prism 8 (GraphPad Software Inc., USA).

### Metabolomic analysis

To compare the relative abundance of DOXP, MEP and IPP/DMAPP in different strains, intracellular tachyzoites of indicated strains were released from host cells by syringe passage, washed with PBS and resuspended (6×10^7^ parasites) with 500 μl of chloroform, methanol, and acetonitrile (2:1:1, v/v/v) [73]. The resulting mixture was subjected to ultrasonication (6 cycles of 10 min, each with 1 min interval). Then 500 μl of ddH_2_O was added and centrifuged at 16000 g for 30 min at 4°C after sufficient shaking. The upper aqueous phase was lyophilized in an ultra-low temperature vacuum and then resuspended in 50 μl ddH_2_O. The extracted metabolites were then analyzed by LC-MS/MS with a 6500 plus QTrap mass spectrometer (AB SCIEX, USA) coupled with ACQUITY UPLC H-Class system (Waters, USA). Chromatographic separation was achieved using an ACQUITY UPLC BEH Amide column (2.1×100 mm,1.7 μm; Waters). Mobile phase A contained HPLC-grade H_2_O-ACN (10/90, v/v) with 10 mM ammonium bicarbonate, and mobile phase B was H_2_O-ACN (50/50, v/v) with 10 mM ammonium bicarbonate. Gradient elution was performed at a flow rate of 0.35 mL/min. The gradient condition was initialized at 15% B held for 2 min, then increased to 95% B for 10 min, and finally returned to 2% B at 12.1 min. The total run time for each sample was 15 min. Data were acquired in the multiple reaction monitor (MRM) mode. The ion transitions were optimized using standard DOXP, MEP and IPP (Sigma–Aldrich, USA) compounds. The nebulizer gas (Gas1), heater gas (Gas2), and curtain gas were set at 55, 55, and 35 psi, respectively. The ion spray voltage was -4500 v for the negative ion mode. The optimal probe temperature was determined to be 500°C, and the column oven temperature was set to 45°C. SCIEX OS 1.6 software was applied for metabolite identification and peak integration. For each experiment, the calculated abundance of each compound in the control group was set as 1 to normalize that in the experimental group. Each strain was tested three times independently.

To examine the fatty acid synthesis activities in the iTPI2 and iGAPDH2 strains, intracellularly tachyzoites with or without 5 days’ rapamycin (iTPI2) or ATc (iGAPDH2) pretreatments were incubated in DMEM medium containing 8 mM [^13^C_6_]-glucose and cultured for 48 hours under the same pretreatment conditions [36]. Then, parasites were released from host cells by needle passage and purified by filtration. The parasites were resuspended in 500 μL ddH_2_O and then transferred to a new glass tube containing 500 μL methanol and 1 mL chloroform. The mixture was vortexed for 1 min and stood for 30 min. After centrifugation at 14000 g for 15 min at 4°C, 800 μL chloroform layer was evaporated to dryness under a nitrogen stream. Then 500 μL of 0.5M KOH (contained 75% ethanol) was added and held at 80°C for 60 min. Then, 100 μL formate and 600 μL n-hexane were added. 500 μL n-hexane layer was evaporated to dryness. The extracted metabolites were mixed with 10 μL dimethyl sulfoxide (DMSO), 1-Hydroxybenzotriazole (HoBt) (in DMSO), 20 μL cholamine (in DMSO with 200 mM Triethylamine (TEA)) and 10 μL 1-[Bis(dimethylamino)methylene]-1H-1,2,3-triazolo[4,5-b] pyridinium 3-oxide hexafluorophosphate (HATU) (in DMSO) and the resulting mixture was incubated at room temperature for 5 min. Then 60 μL acetonitrile was added and centrifugated at 14000 g for 15 min at 4°C prior to UHPLC-HRMS analysis [74]. Chromatographic separation was performed on a Thermo Fisher Ultimate 3000 UHPLC system with a Waters BEH C18 column (2.1 mm × 100 mm, 1.7 μm) (Waters, USA). The injection volume was 1 μL and the flow rate was 0.3 mL/min. The column temperature was 40°C. The mobile phases consisted of water (phase A) and acetonitrile (phase B), both with 0.1% formate. A linear gradient elution was performed with the following program: 0 min, 10%B; 4 min, 30% B; 8min, 45% B; 11 min, 50%B; 14 min, 70%B; 15 min, 100%B and held to 18 min; 18.1 min, 10%B and held to 20 min. The eluents were analyzed on a Thermo Fisher Q Exactive Hybrid Quadrupole-Orbitrap Mass Spectrometry (QE) in Heated Electrospray Ionization Positive (HESI+) mode. Spray voltage was set to 4000 V. Capillary and probe heater temperature were separately 320°C and 350°C. Sheath gas flow rate was 35 Arb (arbitrary unit), and Aux gas flow rate was 10 Arb. S-Lens RF Level was 50 Arb. The full scan was operated at a high-resolution of 70000 FWHM (m/z = 200) at a range of 100–1500 m/z with AGC Target setting at 1×10^6^.

### Statistical analysis

Statistical analyses were performed in Prism 8 using Student’s t-tests or two-way analysis of variance followed by Tukey’s multiple comparison post-tests, as indicated in figure legends. For the parasite replication tests, Tukey’s multiple comparison tests were applied to all comparable datasets (for example, the 1 parasite per PV group of strain A vs the 1 parasite per PV group of strain B; the 2 parasites per PV group of condition A vs the 2 parasites per PV group of condition B). Then, the lowest *P* value was taken to indicate the statistical significance between the two strains/conditions.

## Supporting information

S1 Fig SDS-PAGE assessing the purity and concentration of purified recombinant PGK1 and PGK2 proteins. Bovine serum albumin (BSA) standards at indicated concentrations were included to estimate the concentrations of PGK1 and PGK2.
(PDF)

S2 Fig The cytosolic PGK1 is critical for optimal parasite growth. A, schematic illustration of inserting an anhydrotetracycline (ATc) regulatable promoter pS1O7 upstream the coding sequence of *PGK1* in the TATi line to construct the conditional depletion strain iPGK1. 5H and 3H are homology arms and Ty is an epitope tag. B, diagnostic PCR on an iPGK1 clone. C, Western blotting checking the suppression of PGK1 expression by ATc treatment, through probing Ty that was fused to the N terminus of PGK1 in the iPGK1 strain. ALD was included as a loading control. D, plaque assay comparing the overall growth of indicated strains in the presence or absence of ATc. E, relative sizes of plaques in D, expressed as pixel units. Means ± SEM of more than 100 plaques, ***P < 0.001, student’s t-test. F, intracellular proliferation rates of the TATi and iPGK1 strains under indicated conditions, as determined by replication assays described in Fig 2E. Means ± SEM of three independent experiments, each with three replicates. ***P < 0.001, two-way ANOVA with Tukey’s post-tests.
(PDF)

S3 Fig The iTPI2 strain turned on YFP expression after rapamycin treatment, which indicated TPI2 deletion. Parasites were treated with rapamycin for five days and then imaged under a fluorescence microscope. Nearly 100% of the parasites became YFP positive after rapamycin treatment.
(PDF)

S4 Fig The messenger RNA levels of TPI2 or GAPDH2 before and after gene depletion, as determined by RT-PCR. The iTPI2 and iGAPDH2 mutants were treated with or without rapamycin or ATc for 5 days, respectively. Then, the parasites were collected and the messenger RNA levels of TPI2 or GAPDH2 were quantified by RT-PCR, using β-tubulin as a reference. The relative mRNA level of the target gene in each sample was expressed as fold of the mRNA level of β-tubulin. Means ± SEM of three independent experiments. ***P < 0.001, student’s t-test.
(PDF)

S5 Fig TPI2 depletion does not affect fatty acids synthesis. The iTPI2 strain was pretreated with or without rapamycin for 3 days and then incubated in medium containing 8 mM ^13^C_6_-glucose and cultured under the same pretreatment condition for another two days. Then the parasites were harvested and the incorporation of ^13^C into each fatty acid species was determined by LC-MS. The percentage of fatty acid that contained one or more ^13^C atoms was plotted for each fatty acid species.
(PDF)

S1 Table Plasmids used in this study. (DOCX)

S2 Table Primers used in this study. (DOCX)

S3 Table Raw data of DOXP/MEP/IPP/DMAPP levels measured by MS. (DOCX)

## References

[1] Vekemans J, Schellenberg D, Benns S, O’Brien K, Alonso P. Meeting report: WHO consultation on malaria vaccine development, Geneva, 15–16 July 2019. Vaccine. 2021;39(22):2907–16. doi: 10.1016/j.vaccine.2021.03.093 33931251

[2] Kotloff KL, Nataro JP, Blackwelder WC, Nasrin D, Farag TH, Panchalingam S, et al. Burden and aetiology of diarrhoeal disease in infants and young children in developing countries (the Global Enteric Multicenter Study, GEMS): a prospective, case-control study. Lancet (London, England). 2013;382(9888):209–22. doi: 10.1016/S0140-6736(13)60844-2 23680352

[3] Gold DA, Kaplan AD, Lis A, Bett GC, Rosowski EE, Cirelli KM, et al. The Toxoplasma Dense Granule Proteins GRA17 and GRA23 Mediate the Movement of Small Molecules between the Host and the Parasitophorous Vacuole. Cell host & microbe. 2015;17(5):642–52. doi: 10.1016/j.chom.2015.04.003 25974303 PMC4435723

[4] MacRae JI, Sheiner L, Nahid A, Tonkin C, Striepen B, McConville MJ. Mitochondrial metabolism of glucose and glutamine is required for intracellular growth of Toxoplasma gondii. Cell host & microbe. 2012;12(5):682–92. doi: 10.1016/j.chom.2012.09.013 23159057 PMC3990185

[5] Blume M, Rodriguez-Contreras D, Landfear S, Fleige T, Soldati-Favre D, Lucius R, et al. Host-derived glucose and its transporter in the obligate intracellular pathogen Toxoplasma gondii are dispensable by glutaminolysis. Proceedings of the National Academy of Sciences of the United States of America. 2009;106(31):12998–3003. doi: 10.1073/pnas.0903831106 19617561 PMC2722337

[6] Nitzsche R, Zagoriy V, Lucius R, Gupta N. Metabolic Cooperation of Glucose and Glutamine Is Essential for the Lytic Cycle of Obligate Intracellular Parasite Toxoplasma gondii. The Journal of biological chemistry. 2016;291(1):126–41. doi: 10.1074/jbc.M114.624619 26518878 PMC4697150

[7] Krishnan A, Kloehn J, Lunghi M, Chiappino-Pepe A, Waldman BS, Nicolas D, et al. Functional and Computational Genomics Reveal Unprecedented Flexibility in Stage-Specific Toxoplasma Metabolism. Cell host & microbe. 2020;27(2):290-306.e11. doi: 10.1016/j.chom.2020.01.002 31991093

[8] Shukla A, Olszewski KL, Llinás M, Rommereim LM, Fox BA, Bzik DJ, et al. Glycolysis is important for optimal asexual growth and formation of mature tissue cysts by Toxoplasma gondii. International journal for parasitology. 2018;48(12):955–68. doi: 10.1016/j.ijpara.2018.05.013 30176233

[9] Nitzsche R, Günay-Esiyok Ö, Tischer M, Zagoriy V, Gupta N. A plant/fungal-type phosphoenolpyruvate carboxykinase located in the parasite mitochondrion ensures glucose-independent survival of Toxoplasma gondii. The Journal of biological chemistry. 2017;292(37):15225–39. doi: 10.1074/jbc.M117.802702 28726641 PMC5602384

[10] Shen B, Sibley LD. Toxoplasma aldolase is required for metabolism but dispensable for host-cell invasion. Proceedings of the National Academy of Sciences of the United States of America. 2014;111(9):3567–72. doi: 10.1073/pnas.1315156111 24550496 PMC3948255

[11] Yang X, Yin X, Liu J, Niu Z, Yang J, Shen B. Essential role of pyrophosphate homeostasis mediated by the pyrophosphate-dependent phosphofructokinase in Toxoplasma gondii. PLoS pathogens. 2022;18(2):e1010293. doi: 10.1371/journal.ppat.1010293 35104280 PMC8836295

[12] Xia N, Ye S, Liang X, Chen P, Zhou Y, Fang R, et al. Pyruvate Homeostasis as a Determinant of Parasite Growth and Metabolic Plasticity in Toxoplasma gondii. mBio. 2019;10(3). doi: 10.1128/mBio.00898-19 31186321 PMC6561023

[13] Blume M, Nitzsche R, Sternberg U, Gerlic M, Masters SL, Gupta N, et al. A Toxoplasma gondii Gluconeogenic Enzyme Contributes to Robust Central Carbon Metabolism and Is Essential for Replication and Virulence. Cell host & microbe. 2015;18(2):210–20. doi: 10.1016/j.chom.2015.07.008 26269956

[14] Lunghi M, Galizi R, Magini A, Carruthers VB, Di Cristina M. Expression of the glycolytic enzymes enolase and lactate dehydrogenase during the early phase of Toxoplasma differentiation is regulated by an intron retention mechanism. Molecular microbiology. 2015;96(6):1159–75. doi: 10.1111/mmi.12999 25777509

[15] Xia N, Zhou T, Liang X, Ye S, Zhao P, Yang J, et al. A Lactate Fermentation Mutant of Toxoplasma Stimulates Protective Immunity Against Acute and Chronic Toxoplasmosis. Frontiers in immunology. 2018;9:1814. doi: 10.3389/fimmu.2018.01814 30147689 PMC6096001

[16] Fleige T, Fischer K, Ferguson DJ, Gross U, Bohne W. Carbohydrate metabolism in the Toxoplasma gondii apicoplast: localization of three glycolytic isoenzymes, the single pyruvate dehydrogenase complex, and a plastid phosphate translocator. Eukaryotic cell. 2007;6(6):984–96. doi: 10.1128/EC.00061-07 17449654 PMC1951530

[17] Maeda T, Saito T, Harb OS, Roos DS, Takeo S, Suzuki H, et al. Pyruvate kinase type-II isozyme in Plasmodium falciparum localizes to the apicoplast. Parasitology international. 2009;58(1):101–5. doi: 10.1016/j.parint.2008.10.005 19015045 PMC6157015

[18] Tang Y, Meister TR, Walczak M, Pulkoski-Gross MJ, Hari SB, Sauer RT, et al. A mutagenesis screen for essential plastid biogenesis genes in human malaria parasites. PLoS biology. 2019;17(2):e3000136. doi: 10.1371/journal.pbio.3000136 30726238 PMC6380595

[19] Swift RP, Rajaram K, Keutcha C, Liu HB, Kwan B, Dziedzic A, et al. The NTP generating activity of pyruvate kinase II is critical for apicoplast maintenance in Plasmodium falciparum. eLife. 2020;9. doi: 10.7554/eLife.50807 32815516 PMC7556864

[20] McFadden GI. The apicoplast. Protoplasma. 2011;248(4):641–50. doi: 10.1007/s00709-010-0250-5 21165662

[21] McFadden GI, Yeh E. The apicoplast: now you see it, now you don’t. International journal for parasitology. 2017;47(2–3):137–44. doi: 10.1016/j.ijpara.2016.08.005 27773518 PMC5406208

[22] Banerjee A, Sharkey TD. Methylerythritol 4-phosphate (MEP) pathway metabolic regulation. Natural product reports. 2014;31(8):1043–55. doi: 10.1039/c3np70124g 24921065

[23] Nair SC, Brooks CF, Goodman CD, Sturm A, McFadden GI, Sundriyal S, et al. Apicoplast isoprenoid precursor synthesis and the molecular basis of fosmidomycin resistance in Toxoplasma gondii. The Journal of experimental medicine. 2011;208(7):1547–59. doi: 10.1084/jem.20110039 21690250 PMC3135366

[24] Buhaescu I, Izzedine H. Mevalonate pathway: a review of clinical and therapeutical implications. Clinical biochemistry. 2007;40(9–10):575–84. doi: 10.1016/j.clinbiochem.2007.03.016 17467679

[25] Kadian K, Gupta Y, Singh HV, Kempaiah P, Rawat M. Apicoplast Metabolism: Parasite’s Achilles’ Heel. Current topics in medicinal chemistry. 2018;18(22):1987–97. doi: 10.2174/1568026619666181130134742 30499407

[26] Rodríguez-Concepción M. The MEP pathway: a new target for the development of herbicides, antibiotics and antimalarial drugs. Current pharmaceutical design. 2004;10(19):2391–400. doi: 10.2174/1381612043384006 15279616

[27] Wiesner J, Borrmann S, Jomaa H. Fosmidomycin for the treatment of malaria. Parasitology research. 2003;90 Suppl 2:S71–6. 12937969 10.1007/s00436-002-0770-9

[28] Missinou MA, Borrmann S, Schindler A, Issifou S, Adegnika AA, Matsiegui PB, et al. Fosmidomycin for malaria. Lancet (London, England). 2002;360(9349):1941–2. doi: 10.1016/S0140-6736(02)11860-5 12493263

[29] Waller RF, Keeling PJ, Donald RG, Striepen B, Handman E, Lang-Unnasch N, et al. Nuclear-encoded proteins target to the plastid in Toxoplasma gondii and Plasmodium falciparum. Proceedings of the National Academy of Sciences of the United States of America. 1998;95(21):12352–7. doi: 10.1073/pnas.95.21.12352 9770490 PMC22835

[30] Gornicki P. Apicoplast fatty acid biosynthesis as a target for medical intervention in apicomplexan parasites. International journal for parasitology. 2003;33(9):885–96. doi: 10.1016/s0020-7519(03)00133-4 12906873

[31] Lu JZ, Lee PJ, Waters NC, Prigge ST. Fatty Acid synthesis as a target for antimalarial drug discovery. Combinatorial chemistry & high throughput screening. 2005;8(1):15–26. doi: 10.2174/1386207053328192 15720194

[32] Goodman CD, McFadden GI. Fatty acid biosynthesis as a drug target in apicomplexan parasites. Current drug targets. 2007;8(1):15–30. doi: 10.2174/138945007779315579 17266528

[33] Liu J, Li TT, Liang QL, Elsheikha HM, Zhao DY, Zhang ZW, et al. Characterization of functions in parasite growth and virulence of four Toxoplasma gondii genes involved in lipid synthesis by CRISPR-Cas9 system. Parasitology research. 2021;120(11):3749–59. doi: 10.1007/s00436-021-07308-3 34499198

[34] Xu XP, Elsheikha HM, Liu WG, Zhang ZW, Sun LX, Liang QL, et al. The Role of Type II Fatty Acid Synthesis Enzymes FabZ, ODSCI, and ODSCII in the Pathogenesis of Toxoplasma gondii Infection. Frontiers in microbiology. 2021;12:703059. doi: 10.3389/fmicb.2021.703059 34531837 PMC8438308

[35] Yu M, Kumar TR, Nkrumah LJ, Coppi A, Retzlaff S, Li CD, et al. The fatty acid biosynthesis enzyme FabI plays a key role in the development of liver-stage malarial parasites. Cell host & microbe. 2008;4(6):567–78. 19064257 10.1016/j.chom.2008.11.001 PMC2646117

[36] Liang X, Cui J, Yang X, Xia N, Li Y, Zhao J, et al. Acquisition of exogenous fatty acids renders apicoplast-based biosynthesis dispensable in tachyzoites of Toxoplasma. The Journal of biological chemistry. 2020;295(22):7743–52. doi: 10.1074/jbc.RA120.013004 32341123 PMC7261779

[37] Kloehn J, Harding CR, Soldati-Favre D. Supply and demand-heme synthesis, salvage and utilization by Apicomplexa. The FEBS journal. 2021;288(2):382–404. doi: 10.1111/febs.15445 32530125

[38] Gisselberg JE, Dellibovi-Ragheb TA, Matthews KA, Bosch G, Prigge ST. The suf iron-sulfur cluster synthesis pathway is required for apicoplast maintenance in malaria parasites. PLoS pathogens. 2013;9(9):e1003655. doi: 10.1371/journal.ppat.1003655 24086138 PMC3784473

[39] Swift RP, Rajaram K, Liu HB, Dziedzic A, Jedlicka AE, Roberts AD, et al. A mevalonate bypass system facilitates elucidation of plastid biology in malaria parasites. PLoS pathogens. 2020;16(2):e1008316. doi: 10.1371/journal.ppat.1008316 32059044 PMC7046295

[40] Swift RP, Rajaram K, Liu HB, Prigge ST. Dephospho-CoA kinase, a nuclear-encoded apicoplast protein, remains active and essential after Plasmodium falciparum apicoplast disruption. The EMBO journal. 2021;40(16):e107247. doi: 10.15252/embj.2020107247 34031901 PMC8365264

[41] Brooks CF, Johnsen H, van Dooren GG, Muthalagi M, Lin SS, Bohne W, et al. The toxoplasma apicoplast phosphate translocator links cytosolic and apicoplast metabolism and is essential for parasite survival. Cell host & microbe. 2010;7(1):62–73. doi: 10.1016/j.chom.2009.12.002 20036630 PMC3013619

[42] Ferguson DJ, Parmley SF, Tomavo S. Evidence for nuclear localisation of two stage-specific isoenzymes of enolase in Toxoplasma gondii correlates with active parasite replication. International journal for parasitology. 2002;32(11):1399–410. doi: 10.1016/s0020-7519(02)00129-7 12350375

[43] Jewett TJ, Sibley LD. Aldolase forms a bridge between cell surface adhesins and the actin cytoskeleton in apicomplexan parasites. Molecular cell. 2003;11(4):885–94. doi: 10.1016/s1097-2765(03)00113-8 12718875

[44] Mouveaux T, Oria G, Werkmeister E, Slomianny C, Fox BA, Bzik DJ, et al. Nuclear glycolytic enzyme enolase of Toxoplasma gondii functions as a transcriptional regulator. PloS one. 2014;9(8):e105820. doi: 10.1371/journal.pone.0105820 25153525 PMC4143315

[45] Saito T, Nishi M, Lim MI, Wu B, Maeda T, Hashimoto H, et al. A novel GDP-dependent pyruvate kinase isozyme from Toxoplasma gondii localizes to both the apicoplast and the mitochondrion. The Journal of biological chemistry. 2008;283(20):14041–52. doi: 10.1074/jbc.M709015200 18326043 PMC2376232

[46] Sidik SM, Huet D, Ganesan SM, Huynh MH, Wang T, Nasamu AS, et al. A Genome-wide CRISPR Screen in Toxoplasma Identifies Essential Apicomplexan Genes. Cell. 2016;166(6):1423-35.e12. doi: 10.1016/j.cell.2016.08.019 27594426 PMC5017925

[47] Hunt A, Russell MRG, Wagener J, Kent R, Carmeille R, Peddie CJ, et al. Differential requirements for cyclase-associated protein (CAP) in actin-dependent processes of Toxoplasma gondii. eLife. 2019;8. doi: 10.7554/eLife.50598 31577230 PMC6785269

[48] Wierenga RK, Kapetaniou EG, Venkatesan R. Triosephosphate isomerase: a highly evolved biocatalyst. Cellular and molecular life sciences: CMLS. 2010;67(23):3961–82. doi: 10.1007/s00018-010-0473-9 20694739 PMC11115733

[49] Yeh E, DeRisi JL. Chemical rescue of malaria parasites lacking an apicoplast defines organelle function in blood-stage Plasmodium falciparum. PLoS biology. 2011;9(8):e1001138. doi: 10.1371/journal.pbio.1001138 21912516 PMC3166167

[50] Baumeister S, Wiesner J, Reichenberg A, Hintz M, Bietz S, Harb OS, et al. Fosmidomycin uptake into Plasmodium and Babesia-infected erythrocytes is facilitated by parasite-induced new permeability pathways. PloS one. 2011;6(5):e19334. doi: 10.1371/journal.pone.0019334 21573242 PMC3087763

[51] Clermont S, Corbier C, Mely Y, Gerard D, Wonacott A, Branlant G. Determinants of coenzyme specificity in glyceraldehyde-3-phosphate dehydrogenase: role of the acidic residue in the fingerprint region of the nucleotide binding fold. Biochemistry. 1993;32(38):10178–84. doi: 10.1021/bi00089a038 8399144

[52] Dubey R, Staker BL, Foe IT, Bogyo M, Myler PJ, Ngô HM, et al. Membrane skeletal association and post-translational allosteric regulation of Toxoplasma gondii GAPDH1. Molecular microbiology. 2017;103(4):618–34. doi: 10.1111/mmi.13577 27859784 PMC5296235

[53] Brown KM, Long S, Sibley LD. Conditional Knockdown of Proteins Using Auxin-inducible Degron (AID) Fusions in Toxoplasma gondii. Bio-protocol. 2018;8(4). doi: 10.21769/BioProtoc.2728 29644255 PMC5890294

[54] Amiar S, MacRae JI, Callahan DL, Dubois D, van Dooren GG, Shears MJ, et al. Apicoplast-Localized Lysophosphatidic Acid Precursor Assembly Is Required for Bulk Phospholipid Synthesis in Toxoplasma gondii and Relies on an Algal/Plant-Like Glycerol 3-Phosphate Acyltransferase. PLoS pathogens. 2016;12(8):e1005765. doi: 10.1371/journal.ppat.1005765 27490259 PMC4973916

[55] He CY, Shaw MK, Pletcher CH, Striepen B, Tilney LG, Roos DS. A plastid segregation defect in the protozoan parasite Toxoplasma gondii. The EMBO journal. 2001;20(3):330–9. doi: 10.1093/emboj/20.3.330 11157740 PMC133478

[56] Akuh OA, Elahi R, Prigge ST, Seeber F. The ferredoxin redox system—an essential electron distributing hub in the apicoplast of Apicomplexa. Trends in parasitology. 2022;38(10):868–81. doi: 10.1016/j.pt.2022.08.002 35999149 PMC9481715

[57] Henkel S, Frohnecke N, Maus D, McConville MJ, Laue M, Blume M, et al. Toxoplasma gondii apicoplast-resident ferredoxin is an essential electron transfer protein for the MEP isoprenoid-biosynthetic pathway. The Journal of biological chemistry. 2022;298(1):101468. doi: 10.1016/j.jbc.2021.101468 34896149 PMC8717598

[58] Flügge UI, Häusler RE, Ludewig F, Gierth M. The role of transporters in supplying energy to plant plastids. Journal of experimental botany. 2011;62(7):2381–92. doi: 10.1093/jxb/erq361 21511915

[59] Seeber F, Aliverti A, Zanetti G. The plant-type ferredoxin-NADP+ reductase/ferredoxin redox system as a possible drug target against apicomplexan human parasites. Current pharmaceutical design. 2005;11(24):3159–72. doi: 10.2174/1381612054864957 16178751

[60] Shears MJ, Botté CY, McFadden GI. Fatty acid metabolism in the Plasmodium apicoplast: Drugs, doubts and knockouts. Molecular and biochemical parasitology. 2015;199(1–2):34–50. doi: 10.1016/j.molbiopara.2015.03.004 25841762

[61] Pei Y, Tarun AS, Vaughan AM, Herman RW, Soliman JM, Erickson-Wayman A, et al. Plasmodium pyruvate dehydrogenase activity is only essential for the parasite’s progression from liver infection to blood infection. Molecular microbiology. 2010;75(4):957–71. doi: 10.1111/j.1365-2958.2009.07034.x 20487290

[62] Fast NM, Kissinger JC, Roos DS, Keeling PJ. Nuclear-encoded, plastid-targeted genes suggest a single common origin for apicomplexan and dinoflagellate plastids. Molecular biology and evolution. 2001;18(3):418–26. doi: 10.1093/oxfordjournals.molbev.a003818 11230543

[63] Pino P, Foth BJ, Kwok LY, Sheiner L, Schepers R, Soldati T, et al. Dual targeting of antioxidant and metabolic enzymes to the mitochondrion and the apicoplast of Toxoplasma gondii. PLoS pathogens. 2007;3(8):e115. doi: 10.1371/journal.ppat.0030115 17784785 PMC1959373

[64] Jezewski AJ, Guggisberg AM, Hodge DM, Ghebremichael N, John GN, McLellan LK, et al. GAPDH mediates drug resistance and metabolism in Plasmodium falciparum malaria parasites. PLoS pathogens. 2022;18(9):e1010803. doi: 10.1371/journal.ppat.1010803 36103572 PMC9512246

[65] Saeed S, Tremp AZ, Sharma V, Lasonder E, Dessens JT. NAD(P) transhydrogenase has vital non-mitochondrial functions in malaria parasite transmission. EMBO reports. 2020;21(3):e47832. doi: 10.15252/embr.201947832 31951090 PMC7054674

[66] Meissner M, Brecht S, Bujard H, Soldati D. Modulation of myosin A expression by a newly established tetracycline repressor-based inducible system in Toxoplasma gondii. Nucleic acids research. 2001;29(22):E115. doi: 10.1093/nar/29.22.e115 11713335 PMC92585

[67] Shen B, Brown KM, Lee TD, Sibley LD. Efficient gene disruption in diverse strains of Toxoplasma gondii using CRISPR/CAS9. mBio. 2014;5(3):e01114–14. doi: 10.1128/mBio.01114-14 24825012 PMC4030483

[68] Pan M, Li M, Li L, Song Y, Hou L, Zhao J, et al. Identification of Novel Dense-Granule Proteins in Toxoplasma gondii by Two Proximity-Based Biotinylation Approaches. Journal of proteome research. 2019;18(1):319–30. doi: 10.1021/acs.jproteome.8b00626 30362762

[69] Trost P, Fermani S, Marri L, Zaffagnini M, Falini G, Scagliarini S, et al. Thioredoxin-dependent regulation of photosynthetic glyceraldehyde-3-phosphate dehydrogenase: autonomous vs. CP12-dependent mechanisms. Photosynthesis research. 2006;89(2–3):263–75. doi: 10.1007/s11120-006-9099-z 17031544

[70] Toursel C, Dzierszinski F, Bernigaud A, Mortuaire M, Tomavo S. Molecular cloning, organellar targeting and developmental expression of mitochondrial chaperone HSP60 in Toxoplasma gondii. Molecular and biochemical parasitology. 2000;111(2):319–32. doi: 10.1016/s0166-6851(00)00324-8 11163440

[71] Zheng J, Su W, Cao S, Zhang Z, Du C, Jia H. TgMAP1c is involved in apicoplast biogenesis in Toxoplasma gondii. International journal for parasitology. 2020;50(6–7):487–99. doi: 10.1016/j.ijpara.2020.03.004 32380097

[72] Xia N, Yang J, Ye S, Zhang L, Zhou Y, Zhao J, et al. Functional analysis of Toxoplasma lactate dehydrogenases suggests critical roles of lactate fermentation for parasite growth in vivo. Cellular microbiology. 2018;20(1). doi: 10.1111/cmi.12794 29028143

[73] Zhang B, Watts KM, Hodge D, Kemp LM, Hunstad DA, Hicks LM, et al. A second target of the antimalarial and antibacterial agent fosmidomycin revealed by cellular metabolic profiling. Biochemistry. 2011;50(17):3570–7. doi: 10.1021/bi200113y 21438569 PMC3082593

[74] Bian X, Sun B, Zheng P, Li N, Wu JL. Derivatization enhanced separation and sensitivity of long chain-free fatty acids: Application to asthma using targeted and non-targeted liquid chromatography-mass spectrometry approach. Analytica chimica acta. 2017;989:59–70. doi: 10.1016/j.aca.2017.08.009 28915943

