# Supplementary material for: Two apicoplast dwelling glycolytic enzymes provide key substrates for metabolic pathways in the apicoplast and are critical for Toxoplasma growth
Source: PLoS Pathog. 2022 Nov 30;18(11):e1011009. doi: 10.1371/journal.ppat.1011009 (PMC9744290; doi:10.1371/journal.ppat.1011009)
Supplement: S1 Fig — Bovine serum albumin (BSA) standards at indicated concentrations were included to estimate the concentrations of PGK1 and PGK2. (PDF) [file ppat.1011009.s001.pdf]

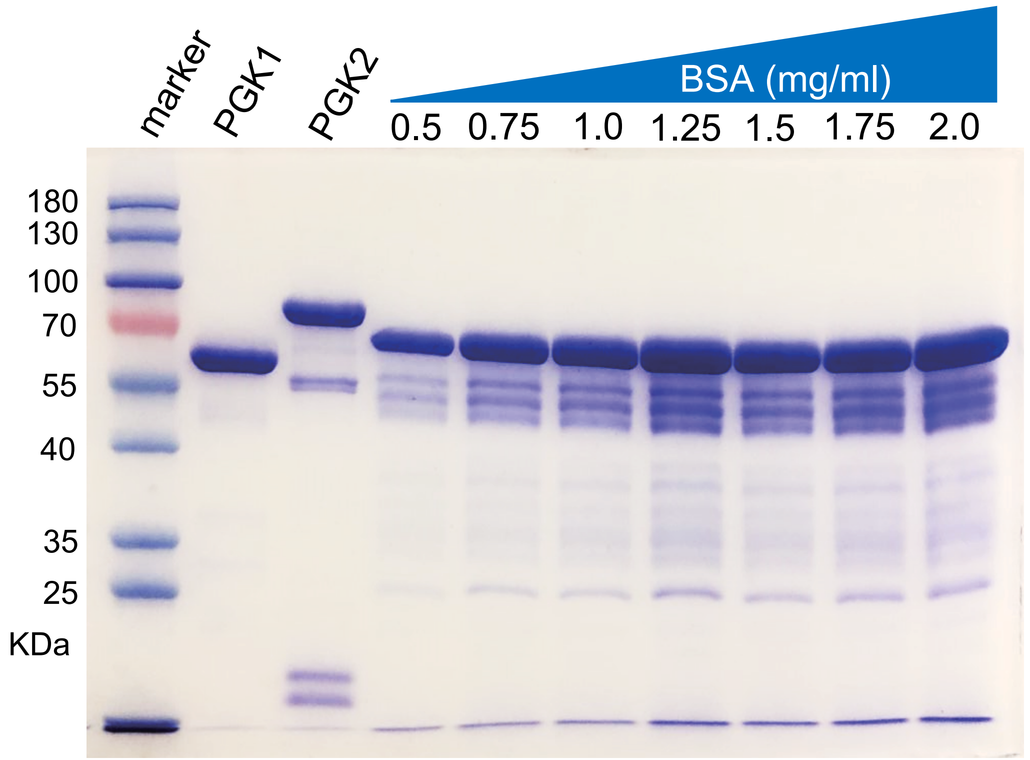

Figure S1. SDS-PAGE assessing the purity and concentration of purified recombinant PGK1 and PGK2 proteins. Bovine serum albumin (BSA) standards at indicated concentrations were included to estimate the concentrations of PGK1 and PGK2.
