# Supplementary material for: Two apicoplast dwelling glycolytic enzymes provide key substrates for metabolic pathways in the apicoplast and are critical for Toxoplasma growth
Source: PLoS Pathog. 2022 Nov 30;18(11):e1011009. doi: 10.1371/journal.ppat.1011009 (PMC9744290; doi:10.1371/journal.ppat.1011009)
Supplement: S2 Fig — A, schematic illustration of inserting an anhydrotetracycline (ATc) regulatable promoter pS1O7 upstream the coding sequence of PGK1 in the TATi line to construct the conditional depletion strain iPGK1. 5H and 3H are homology arms and Ty is an epitope tag. B, diagnostic PCR on an iPGK1 clone. C, Western blotting checking the suppression of PGK1 expression by ATc treatment, through probing Ty that was fused to the N terminus of PGK1 in the iPGK1 strain. ALD was included as a loading control. D, plaque assay comparing the overall growth of indicated strains in the presence or absence of ATc. E, relative sizes of plaques in D, expressed as pixel units. Means ± SEM of more than 100 plaques, ***P < 0.001, student’s t-test. F, intracellular proliferation rates of the TATi and iPGK1 strains under indicated conditions, as determined by replication assays described in Fig 2E. Means ± SEM of three independent experiments, each with three replicates. ***P < 0.001, two-way ANOVA with Tukey’s post-tests. (PDF) [file ppat.1011009.s002.pdf]

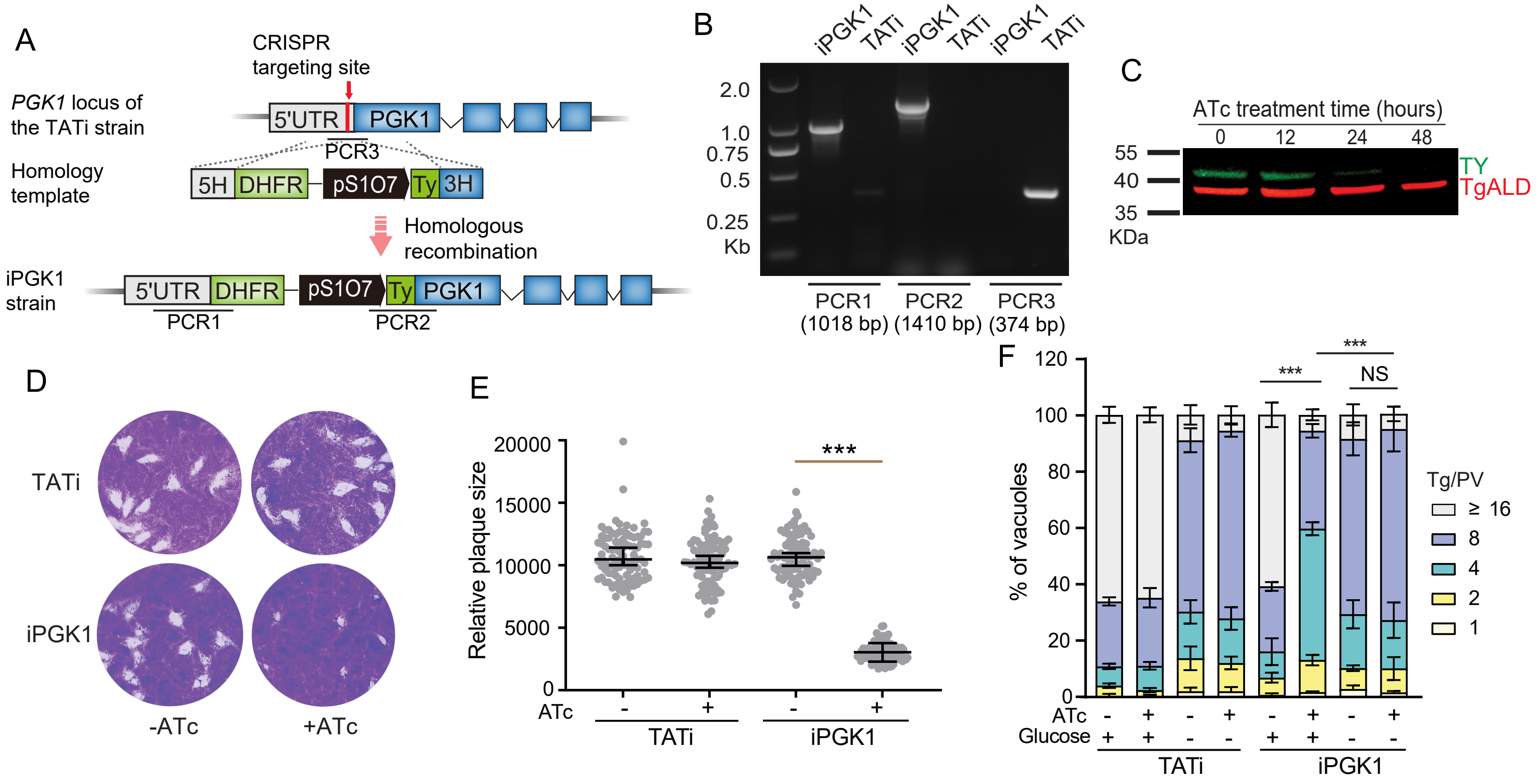

Fig S2. The cytosolic PGK1 is critical for optimal parasite growth. A, schematic illustration of inserting an anhydrotetracycline (ATc) regulatable promoter pS1O7 upstream the coding sequence of *PGK1* in the TATi line to construct the conditional depletion strain iPGK1. 5H and 3H are homology arms and Ty is an epitope tag. B, diagnostic PCR on an iPGK1 clone. C, Western blotting checking the suppression of PGK1 expression by ATc treatment, through probing Ty that was fused to the N terminus of PGK1 in the iPGK1 strain. ALD was included as a loading control. D, plaque assay comparing the overall growth of indicated strains in the presence or absence of ATc. E, relative sizes of plaques in D, expressed as pixel units. Means ± SEM of more than 100 plaques, ****P* < 0.001, student's t-test. F, intracellular proliferation rates of the TATi and iPGK1 strains under indicated conditions, as determined by replication assays described in Fig 2E. Means ± SEM of three independent experiments, each with three replicates. ****P* < 0.001, two-way ANOVA with Tukey's post-tests.
