# Supplementary material for: Two apicoplast dwelling glycolytic enzymes provide key substrates for metabolic pathways in the apicoplast and are critical for Toxoplasma growth
Source: PLoS Pathog. 2022 Nov 30;18(11):e1011009. doi: 10.1371/journal.ppat.1011009 (PMC9744290; doi:10.1371/journal.ppat.1011009)
Supplement: S3 Fig — Parasites were treated with rapamycin for five days and then imaged under a fluorescence microscope. Nearly 100% of the parasites became YFP positive after rapamycin treatment. (PDF) [file ppat.1011009.s003.pdf]

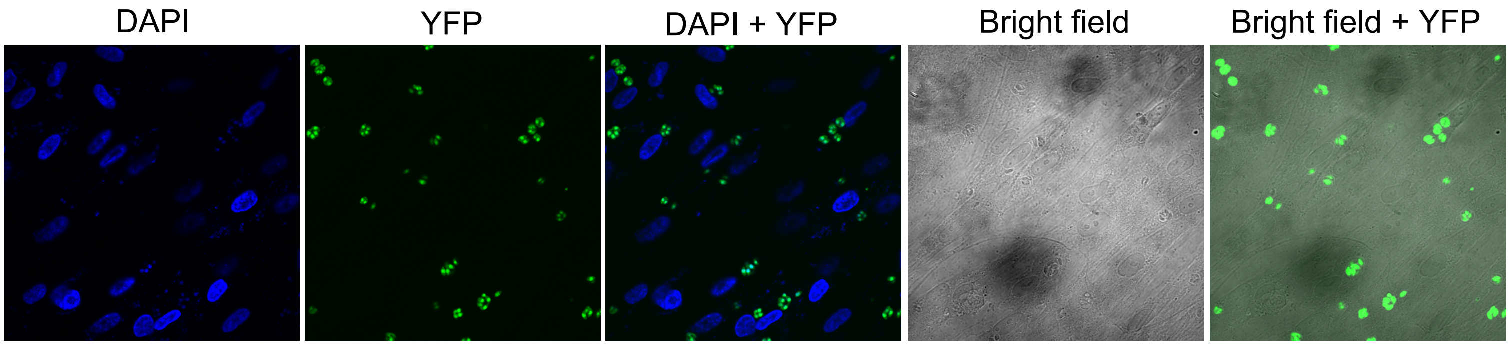

Fig S3. The iTPI2 strain turned on YFP expression after rapamycin treatment, which indicated TPI2 deletion. Parasites were treated with rapamycin for five days and then imaged under a fluorescence microscope. Nearly 100% of the parasites became YFP positive after rapamycin treatment.
