# Supplementary material for: Two apicoplast dwelling glycolytic enzymes provide key substrates for metabolic pathways in the apicoplast and are critical for Toxoplasma growth
Source: PLoS Pathog. 2022 Nov 30;18(11):e1011009. doi: 10.1371/journal.ppat.1011009 (PMC9744290; doi:10.1371/journal.ppat.1011009)
Supplement: S4 Fig — The iTPI2 and iGAPDH2 mutants were treated with or without rapamycin or ATc for 5 days, respectively. Then, the parasites were collected and the messenger RNA levels of TPI2 or GAPDH2 were quantified by RT-PCR, using β-tubulin as a reference. The relative mRNA level of the target gene in each sample was expressed as fold of the mRNA level of β-tubulin. Means ± SEM of three independent experiments. ***P < 0.001, student’s t-test. (PDF) [file ppat.1011009.s004.pdf]

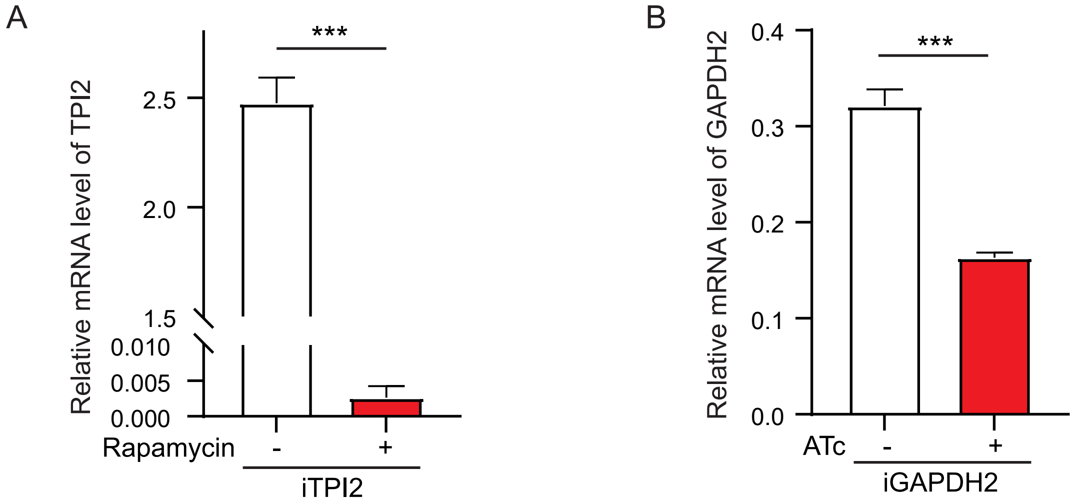

Fig S4. The messenger RNA levels of TPI2 or GAPDH2 before and after gene depletion, as determined by RT-PCR. The iTPI2 and iGAPDH2 mutants were treated with or without rapamycin or ATc for 5 days, respectively. Then, the parasites were collected and the messenger RNA levels of TPI2 or GAPDH2 were quantified by RT-PCR, using β-tubulin as a reference. The relative mRNA level of the target gene in each sample was expressed as fold of the mRNA level of β-tubulin. Means ± SEM of three independent experiments. ****P* < 0.001, student's t-test.
