# Supplementary material for: Two apicoplast dwelling glycolytic enzymes provide key substrates for metabolic pathways in the apicoplast and are critical for Toxoplasma growth
Source: PLoS Pathog. 2022 Nov 30;18(11):e1011009. doi: 10.1371/journal.ppat.1011009 (PMC9744290; doi:10.1371/journal.ppat.1011009)
Supplement: S5 Fig — The iTPI2 strain was pretreated with or without rapamycin for 3 days and then incubated in medium containing 8 mM 13C6-glucose and cultured under the same pretreatment condition for another two days. Then the parasites were harvested and the incorporation of 13C into each fatty acid species was determined by LC-MS. The percentage of fatty acid that contained one or more 13C atoms was plotted for each fatty acid species. (PDF) [file ppat.1011009.s005.pdf]

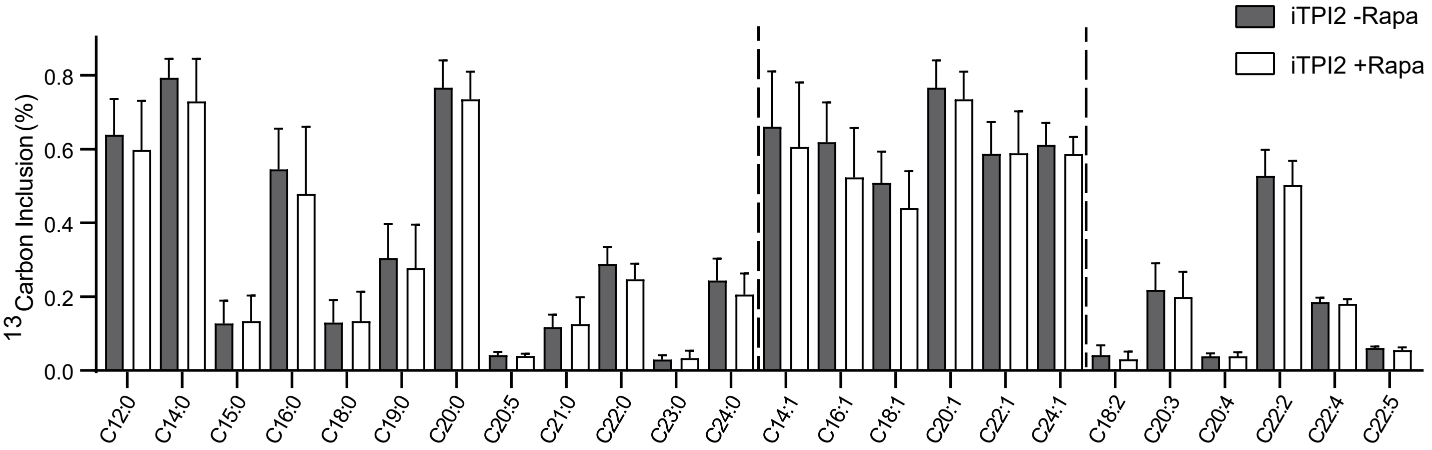

Fig S5. TPI2 depletion does not affect fatty acids synthesis. The iTPI2 strain was pretreated with or without rapamycin for 3 days and then incubated in medium containing 8 mM $^{13}C_6$-glucose and cultured under the same pretreatment condition for another two days. Then the parasites were harvested and the incorporation of ^{13}C into each fatty acid species was determined by LC-MS. The percentage of fatty acid that contained one or more ^{13}C atoms was plotted for each fatty acid species.
