# Supplementary material for: Two apicoplast dwelling glycolytic enzymes provide key substrates for metabolic pathways in the apicoplast and are critical for Toxoplasma growth
Source: PLoS Pathog. 2022 Nov 30;18(11):e1011009. doi: 10.1371/journal.ppat.1011009 (PMC9744290; doi:10.1371/journal.ppat.1011009)
Supplement: S1 Table — (DOCX) [file ppat.1011009.s006.docx]

**Table S1 Plasmids used in this study**

| Name of Plasmid | Genotype/description | Use |
| --- | --- | --- |
| pSAG1‐Cas9‐U6: sgGPI-localization | Expression of CAS9 by a SAG1 promoter and CRISPR sgRNA targeting 3'UTR of *GPI* from a U6 promoter. | CRISPR plasmid targeting 3'UTR of *GPI*, for the construction of GPI-localization strain. |
| pSAG1‐Cas9‐U6: sgPFK1-localization | Expression of CAS9 by a SAG1 promoter and CRISPR sgRNA targeting 3'UTR of *PFK1* from a U6 promoter. | CRISPR plasmid targeting 3'UTR of PFK1, for the construction of PFK1-localization strain. |
| pSAG1‐Cas9‐U6: sgPFK2-localization | Expression of CAS9 by a SAG1 promoter and CRISPR sgRNA targeting 3'UTR of *PFK2* from a U6 promoter. | CRISPR plasmid targeting 3'UTR of PFK2, for the construction of PFK2-localization strain. |
| pSAG1‐Cas9‐U6: sgTPI1-localization | Expression of CAS9 by a SAG1 promoter and CRISPR sgRNA targeting 3'UTR of *TPI1* from a U6 promoter. | CRISPR plasmid targeting 3'UTR of TPI1, for the construction of TPI1-localization strain. |
| pSAG1‐Cas9‐U6: sgTPI2-localization | Expression of CAS9 by a SAG1 promoter and CRISPR sgRNA targeting 3'UTR of *TPI2* from a U6 promoter. | CRISPR plasmid targeting 3'UTR of TPI2, for the construction of TPI2-localization strain. |
| pSAG1‐Cas9‐U6: sgGAPDH1-localization | Expression of CAS9 by a SAG1 promoter and CRISPR sgRNA targeting 3'UTR of *GAPDH1* from a U6 promoter. | CRISPR plasmid targeting 3'UTR of GAPDH1, for the construction of GAPDH1-localization strain. |
| pSAG1‐Cas9‐U6: sgGAPDH2-localization | Expression of CAS9 by a SAG1 promoter and CRISPR sgRNA targeting 3'UTR of *GAPDH2* from a U6 promoter. | CRISPR plasmid targeting 3'UTR of GAPDH2, for the construction of GAPDH2-localization strain. |
| pSAG1‐Cas9‐U6: sgPGK1-localization | Expression of CAS9 by a SAG1 promoter and CRISPR sgRNA targeting 3'UTR of *PGK1* from a U6 promoter. | CRISPR plasmid targeting 3'UTR of PGK1, for the construction of PGK1-localization strain. |
| pSAG1‐Cas9‐U6: sgPGK2-localization | Expression of CAS9 by a SAG1 promoter and CRISPR sgRNA targeting 3'UTR of *PGK2* from a U6 promoter. | CRISPR plasmid targeting 3'UTR of PGK2, for the construction of PGK2-localization strain. |
| pSAG1‐Cas9‐U6: sgPGM1-localization | Expression of CAS9 by a SAG1 promoter and CRISPR sgRNA targeting 3'UTR of *PGM1* from a U6 promoter. | CRISPR plasmid targeting 3'UTR of PGM1, for the construction of PGM1-localization strain. |
| pSAG1‐Cas9‐U6: sgPGM2-localization | Expression of CAS9 by a SAG1 promoter and CRISPR sgRNA targeting 3'UTR of *PGM2* from a U6 promoter. | CRISPR plasmid targeting 3'UTR of PGM2, for the construction of PGM2-localization strain. |
| pSAG1‐Cas9‐U6: sgPGM3-localization | Expression of CAS9 by a SAG1 promoter and CRISPR sgRNA targeting 3'UTR of *PGM3* from a U6 promoter. | CRISPR plasmid targeting 3'UTR of PGM3, for the construction of PGM3-localization strain. |
| pSAG1‐Cas9‐U6: sgPYK1-localization | Expression of CAS9 by a SAG1 promoter and CRISPR sgRNA targeting 3'UTR of *PYK1* from a U6 promoter. | CRISPR plasmid targeting 3'UTR of PYK1, for the construction of PYK1-localization strain. |
| pSAG1‐Cas9‐U6: sgPYK2-localization | Expression of CAS9 by a SAG1 promoter and CRISPR sgRNA targeting 3'UTR of *PYK2* from a U6 promoter. | CRISPR plasmid targeting 3'UTR of PYK2, for the construction of PYK2-localization strain. |
| pPUC19-Ty-3’UTR-DHFR | Template plasmid containing the Ty tag and DHFR drug selection marker. | Homology template for the construction of Ty tagged localization strains HK, PFK2, PGM2, TIP2, GAPDH2, PGK2, PYK2 |
| pSL24m-Linker-smFP-DHFR-LoxP-T7 | Template plasmid containing smHA tag and DHFR drug selection marker. | Homology template for the construction of smHA tagged localization strains GPI, PFK1, TPI1, GAPDH1, PGK1, PGK1, PGM1, PYK1, PGM3 |
| pSAG1‐Cas9‐U6: sgUPRT | Expression of CAS9 driven by a SAG1 promoter and CRISPR sgRNA targeting *UPRT* from a U6 promoter. From reference [1] | Template for gene-specific CRISPR plasmid construction, and the construction of iTPI2compMVA strain |
| pSAG1‐Cas9‐U6: gRNA1-sgTPI1 | Expression of CAS9 by a SAG1 promoter and CRISPR sgRNA targeting 5'UTR of *TPI1* from a U6 promoter. | CRISPR plasmid targeting 5'UTR of *TPI1*, provide the vector for the construction of pSAG1‐Cas9‐U6: double-sgTPI1 |
| pSAG1‐Cas9‐U6: gRNA2-sgTPI1 | Expression of CAS9 by a SAG1 promoter and CRISPR sgRNA targeting 3'UTR of *TPI1* from a U6 promoter. | CRISPR plasmid targeting 3'UTR of *TPI1*, homology template of U6-gRNA2 for the construction of pSAG1‐Cas9‐U6: double-sgTPI1 |
| pSAG1‐Cas9‐U6: double-sgTPI1 | Expression of CAS9 by a SAG1 promoter and CRISPR sgRNA targeting 5'UTR and 3'UTR of *TPI1* from a U6 promoter. | CRISPR plasmid targeting 5'UTR and 3'UTR of *TPI1*, for the construction of DiCre-iTPI1 strain. |
| pSAG1‐Cas9‐U6: gRNA1-sgTPI2 | Expression of CAS9 by a SAG1 promoter and CRISPR sgRNA targeting 5'UTR of *TPI2* from a U6 promoter. | CRISPR plasmid targeting 5'UTR of *TPI2*, provide the vector for the construction of pSAG1‐Cas9‐U6: double-sgTPI2 |
| pSAG1‐Cas9‐U6: gRNA2-sgTPI2 | Expression of CAS9 by a SAG1 promoter and CRISPR sgRNA targeting 3'UTR of *TPI2* from a U6 promoter. | CRISPR plasmid targeting 3'UTR of *TPI2*, homology template of U6-gRNA2 for the construction of pSAG1‐Cas9‐U6: double-sgTPI2 |
| pSAG1‐Cas9‐U6: double-sgTPI2 | Expression of CAS9 by a SAG1 promoter and CRISPR sgRNA targeting 5'UTR and 3'UTR of *TPI2* from a U6 promoter. | CRISPR plasmid targeting 5'UTR and 3'UTR of *TPI2*, for the construction of DiCre-iTPI2 strain. |
| pSAG1‐Cas9‐U6: gRNA1-sgGAPDH2 | Expression of CAS9 by a SAG1 promoter and CRISPR sgRNA targeting 5'UTR of *GAPDH2* from a U6 promoter. | CRISPR plasmid targeting 5'UTR of *GAPDH2*, provide the vector for the construction of pSAG1‐Cas9‐U6: double-sgGAPDH2 |
| pSAG1‐Cas9‐U6: gRNA2-sgGAPDH2 | Expression of CAS9 by a SAG1 promoter and CRISPR sgRNA targeting 3'UTR of *GAPDH2* from a U6 promoter. | CRISPR plasmid targeting 3'UTR of *GAPDH2*, homology template of U6-gRNA2 for the construction of pSAG1‐Cas9‐U6: double-sgGAPDH2 |
| pSAG1‐Cas9‐U6: double-sgGAPDH2 | Expression of CAS9 by a SAG1 promoter and CRISPR sgRNA targeting 5'UTR and 3'UTR of *GAPDH2* from a U6 promoter. | CRISPR plasmid targeting 5'UTR and 3'UTR of *GAPDH2*, for the construction of TATi-iGAPDH2 strain. |
| pSAG1‐Cas9‐U6: sgPGK1 | Expression of CAS9 by a SAG1 promoter and CRISPR sgRNA targeting 5'UTR of *PGK1* from a U6 promoter. | CRISPR plasmid targeting 5'UTR of *PGK1*, for the construction of TATi-iPGK1 strain. |
| pSAG1‐Cas9‐U6: sgPYK2 | Expression of CAS9 by a SAG1 promoter and CRISPR sgRNA targeting *PYK2* from a U6 promoter. | CRISPR plasmid targeting *PYK2*, for the construction of *Δpgk2Δpyk2* strain. |
| pSAG1‐Cas9‐U6: sgPGK2 | Expression of CAS9 by a SAG1 promoter and CRISPR sgRNA targeting *PGK2* from a U6 promoter. | CRISPR plasmid targeting *PGK2*, for the construction of *Δpgk2* strain. |
| pET-28a | Prokaryotic expression plasmids with kana^+^ resistance | Amplification of linearized vector for pET-28a-PGK2-truncation construction |
| pET-28a-PGK2-truncation | PGK2(100-307aa) fused to His tag driven by a T7 promoter with kana^+^ resistance | Prokaryotic expression of the PGK2(100-307) protein |
| pET-28a-2HIS-SUMO | Prokaryotic expression plasmids with kana^+^ resistance | Amplification of SUMO linearized vector for pET-28a-2HIS-SUMO-PGK1 and pET-28a-2HIS-SUMO-PGK2 construction |
| pET-28a-2HIS-SUMO-PGK1 | PGK1 fused to 2×His and SUMO tag driven by a T7 promoter with kana^+^ resistance | Prokaryotic expression of the PGK1 protein |
| pET-28a-2HIS-SUMO-PGK2 | PGK2 fused to 2×His and SUMO tag driven by a T7 promoter with kana^+^ resistance | Prokaryotic expression of the PGK2 (full length) protein |
| p15-MEV-ORF | Contain synthetic sequences of the 4 genes (MVK, PMK, MVD, IDI) of the MVA pathway. *Full sequence of the synthetic genes is listed at the end of this table. | Template for amplification of the CDS of MVA  for pcompMVA-HA-HX-UPRT construction |
| pG265-pTub-UPRT | Contains *UPRT* homology arms, allowing expression of target genes under a tubulin promoter at the *UPRT* locus. Also contain *HXGPRT* cassette. | Template for amplification of the vector for pcompMVA-HA-HX-UPRT construction |
| pcompMVA-HA-HX-UPRT | Expression of MVK-PMK-IDI-MVD-HA by a tubulin promoter | Homology template for the construction of DiCre-iTPI2compMVA strain |
| p7TetOS1 | pS1O7 containing plasmid, Amp resistance, from reference [2] | Template for pSAG1-TetO7 for pPGK1-teto7 and pGAPDH2-teto7 construction |
| pPGK1-teto7 | pS1O7 with the homology arms of PGK1 | Homology template for the construction of TATi-iPGK1 strain |
| pGAPDH2-teto7 | pS1O7-GAPDH2-ty with the homology arms of GAPDH2 | Homology template for the construction of TATi-iGAPDH2 strain |
| pDONR-G265 | Expression of loxp -Ty-loxp-YFP driven by a tubulin promoter. from reference [3] | Template for pDiCre-iTPI1 and pDiCre-iTPI2 construction |
| pDiCre-TPI1 | Expression of loxp-TPI1-Ty-loxp-YFP driven by a tubulin promoter | Homology template for the construction of DiCre-iTPI1 strain |
| pDiCre-TPI2 | Expression of loxp-TPI2-Ty-loxp-YFP driven by a tubulin promoter | Homology template for the construction of DiCre-iTPI2 strain |

*** Sequence of the synthetic genes of the MVA pathway:**

ATGACGAAGAAGGTGGGCGTGGGCCAGGCACACTCTAAGATCATCCTCATCGGCGAACACGCAGTCGTCTACGGCTACCCTGCCATCTCTCTCCCGCTCCTCGAGGTCGAAGTCACGTGCAAAGTCGTCCCGGCGGAGTCTCCATGGAGACTCTACGAGGAGGACACGCTGAGCATGGCGGTCTACGCATCTCTCGAGTACCTCAACATCACAGAAGCGTGCATCCGCTGCGAAATCGACTCTGCCATCCCGGAGAAGCGCGGAATGGGATCTTCGGCAGCCATCTCTATCGCCGCGATCAGAGCGGTCTTCGACTACTACCAGGCCGACCTGCCTCACGACGTCCTCGAAATCCTGGTGAACCGCGCGGAGATGATTGCGCACATGAACCCTTCTGGACTCGACGCCAAGACGTGCCTCTCGGACCAGCCTATCCGCTTCATCAAGAACGTCGGCTTCACGGAACTGGAGATGGACCTCAGCGCGTACCTCGTGATTGCGGACACAGGCGTCTACGGCCACACGAGAGAAGCGATCCAGGTCGTCCAGAACAAAGGCAAGGACGCGCTCCCGTTCCTCCACGCACTCGGAGAACTCACACAGCAGGCGGAGATCGCGATCAGCCAGAAGGACGCAGAAGGCCTCGGACAGATCCTCTCTCAGGCCCACCTCCACCTCAAGGAGATCGGAGTCTCTTCCCTCGAAGCGGACTCTCTCGTCGAGACAGCGCTCTCTCACGGCGCACTCGGAGCAAAAATGTCTGGAGGCGGCCTCGGCGGATGCATTATCGCACTCGTCACGAACCTCACGCACGCGCAGGAACTCGCAGAGAGACTCGAGGAGAAAGGCGCGGTCCAGACGTGGATTGAGTCGCTCGGATCTGGCTCTGGCACGTCTATGGCGCCACTCGGAGGAGCACCTAGACTCGTCCTGCTCTTCTCTGGAAAGCGCAAATCTGGAAAAGACTTCGTCACAGAGGCGCTGCAGTCTCGCCTGGGAGCAGACGTCTGCGCAGTCCTCAGACTCTCTGGACCGCTCAAAGAACAGTACGCGCAGGAGCACGGACTCAACTTCCAGAGACTGCTCGACACAAGCACGTACAAAGAGGCCTTCCGCAAGGACATGATCAGATGGGGCGAAGAAAAGAGACAGGCGGACCCGGGATTCTTCTGCAGAAAGATCGTCGAGGGCATCTCGCAGCCAATCTGGCTCGTCTCTGACACGAGACGCGTCTCGGACATCCAGTGGTTCAGAGAGGCGTACGGAGCGGTCACGCAGACAGTCAGAGTCGTGGCACTCGAGCAGTCTCGCCAGCAGAGAGGATGGGTGTTCACCCCTGGCGTCGACGACGCAGAAAGCGAATGCGGACTCGACAACTTCGGAGACTTCGACTGGGTCATCGAGAACCACGGAGTGGAGCAGCGCCTGGAAGAGCAGCTCGAAAACCTCATCGAGTTCATCCGCTCTAGACTGGGCACGGGCTCTGGAAGAACAATGCAGACGGAGCACGTGATCCTCCTCAACGCGCAGGGAGTGCCTACAGGAACACTGGAAAAGTACGCCGCCCACACGGCGGACACAAGACTCCACCTCGCATTCTCTAGCTGGCTCTTCAACGCGAAGGGCCAGCTCCTCGTCACAAGAAGAGCGCTCTCTAAGAAGGCCTGGCCGGGAGTCTGGACGAACTCTGTCTGCGGACACCCTCAGCTCGGAGAGTCTAACGAGGACGCGGTCATCAGAAGATGCAGATACGAGCTCGGAGTCGAGATCACGCCGCCAGAATCTATCTACCCGGACTTCAGATACCGCGCGACGGACCCTTCTGGCATTGTGGAAAACGAGGTCTGCCCTGTCTTCGCGGCGAGAACAACGTCTGCGCTCCAGATCAACGACGACGAGGTCATGGACTACCAGTGGTGCGACCTCGCGGACGTCCTCCACGGAATTGACGCAACACCGTGGGCGTTCTCTCCGTGGATGGTCATGCAGGCCACAAACCGCGAGGCCAGAAAAAGACTGAGCGCGTTCACACAGCTCAAGAGCGGATCTGGCTCGGGCCCTATGGACAGAGAACCGGTCACGGTCCGCTCTTACGCCAACATCGCGATCATCAAGTACTGGGGCAAGAAGAAAGAAAAAGAGATGGTCCCTGCAACATCTTCTATCAGCCTCACGCTCGAGAACATGTACACAGAGACAACGCTCAGCCCTCTCCCGGCAAACGTCACAGCAGACGAGTTCTACATCAACGGCCAGCTGCAGAACGAGGTCGAACACGCAAAGATGTCTAAAATCATTGACCGCTACCGCCCGGCGGGAGAAGGATTCGTCAGAATTGACACGCAGAACAACATGCCGACGGCGGCAGGACTCTCTTCCTCTTCTTCTGGCCTCTCTGCGCTCGTCAAGGCGTGCAACGCATACTTCAAACTCGGACTGGACCGCTCTCAGCTCGCGCAGGAAGCAAAATTCGCCTCTGGATCTTCTTCTCGCTCTTTCTACGGCCCGCTCGGCGCATGGGACAAAGACTCTGGAGAAATCTACCCGGTGGAGACCGACCTCAAGCTCGCTATGATCATGCTCGTCCTCGAGGACAAAAAGAAGCCTATCTCGTCTCGCGACGGCATGAAGCTCTGCGTGGAAACGTCTACAACATTCGACGACTGGGTGCGCCAGAGCGAAAAAGACTACCAGGACATGCTGATCTACCTGAAAGAGAACGACTTCGCGAAGATCGGCGAGCTGACGGAAAAGAACGCGCTCGCAATGCACGCGACGACGAAGACAGCCTCTCCGGCATTCTCTTACCTCACAGACGCGTCTTACGAAGCCATGGACTTCGTCAGACAGCTCAGAGAGAAAGGAGAGGCATGCTACTTCACGATGGACGCGGGACCGAACGTCAAAGTCTTCTGCCAGGAGAAGGACCTCGAACACCTCTCGGAGATCTTCGGACAGCGCTACCGCCTCATCGTGAGCAAGACGAAAGACCTCTCGCAGGACGACTGCTGCGCCTCTAACGCAAACCCGAACGCCAACCCCAACGCGAACCCT

**References:**

1. Shen B, Brown KM, Lee TD, Sibley LD. Efficient gene disruption in diverse strains of Toxoplasma gondii using CRISPR/CAS9. mBio. 2014;5(3):e01114-14.

2. Meissner M, Brecht S, Bujard H, Soldati D. Modulation of myosin A expression by a newly established tetracycline repressor-based inducible system in Toxoplasma gondii. Nucleic acids research. 2001;29(22):E115.

3. Hunt A, Russell MRG, Wagener J, Kent R, Carmeille R, Peddie CJ, et al. Differential requirements for cyclase-associated protein (CAP) in actin-dependent processes of Toxoplasma gondii. eLife. 2019;8.
