# Supplementary material for: Two apicoplast dwelling glycolytic enzymes provide key substrates for metabolic pathways in the apicoplast and are critical for Toxoplasma growth
Source: PLoS Pathog. 2022 Nov 30;18(11):e1011009. doi: 10.1371/journal.ppat.1011009 (PMC9744290; doi:10.1371/journal.ppat.1011009)
Supplement: S2 Table — (DOCX) [file ppat.1011009.s007.docx]

**Table S2. Primers used in this study**

| Name | Sequence (5′-3′) | Use |
| --- | --- | --- |
| gRNA-GPI-loc-Fw | GCTCAGTCACGTCAGGAATGGTTTTAGAGCTAGAAATAGC | Amplification of CRISPR plasmid vector containing gRNA sequence for TgGPI for pSAG1‐Cas9‐U6: sgGPI-localization construction |
| gRNA‐GPI-loc-Rv | AACTTGACATCCCCATTTAC |  |
| gRNA-HK-loc-Fw | GGCGACACCAGTTGCTTCATGTTTTAGAGCTAGAAATAGC | Amplification of CRISPR plasmid vector containing gRNA sequence for TgHK for pSAG1‐Cas9‐U6: sgHK-localization construction |
| gRNA-HK-loc‐Rv | AACTTGACATCCCCATTTAC |  |
| gRNA-PFK1-loc-Fw | GGACCCTTGCAGATACACGGGTTTTAGAGCTAGAAATAGC | Amplification of CRISPR plasmid vector containing gRNA sequence for TgPFK1 for pSAG1‐Cas9‐U6: sgPFK1-localization construction |
| gRNA-PFK1-loc‐Rv | AACTTGACATCCCCATTTAC |  |
| gRNA-PFK2-loc-Fw | GAGTTTGGAATTCTCTGTCGGTTTTAGAGCTAGAAATAGC | Amplification of CRISPR plasmid vector containing gRNA sequence for TgPFK2 for pSAG1‐Cas9‐U6: sgPFK2-localization construction |
| gRNA-PFK2-loc‐Rv | AACTTGACATCCCCATTTAC |  |
| gRNA-TPI1-loc-Fw | GCTCAGTCACGTCAGGAATGGTTTTAGAGCTAGAAATAGC | Amplification of CRISPR plasmid vector containing gRNA sequence for TgTPI1 for pSAG1‐Cas9‐U6: sgTPI1-localization construction |
| gRNA-TPI1-loc‐Rv | AACTTGACATCCCCATTTAC |  |
| gRNA-TPI2-loc-Fw | GCACAGCCCGCTGAGACAGAGTTTTAGAGCTAGAAATAGC | Amplification of CRISPR plasmid vector containing gRNA sequence for TgTPI2 for pSAG1‐Cas9‐U6: sgTPI2-localization construction |
| gRNA-TPI2-loc‐Rv | AACTTGACATCCCCATTTAC |  |
| gRNA-GAPDH1-loc-Fw | GTTGATTTATGGACACCTAGGTTTTAGAGCTAGAAATAGC | Amplification of CRISPR plasmid vector containing gRNA sequence for TgGAPDH1 for pSAG1‐Cas9‐U6: sgGAPDH1-localization construction |
| gRNA-GAPDH1-loc‐Rv | AACTTGACATCCCCATTTAC |  |
| gRNA-GAPDH2- loc-Fw | GTCAAGTATTAAGCCAGCACGTTTTAGAGCTAGAAATAGC | Amplification of CRISPR plasmid vector containing gRNA sequence for TgGAPDH2 for pSAG1‐Cas9‐U6: sgGAPDH2-localization construction |
| gRNA-GAPDH2- loc‐Rv | AACTTGACATCCCCATTTAC |  |
| gRNA-PGK1- loc-Fw | GCGTAGAGACGCGTCGTGGGGTTTTAGAGCTAGAAATAGC | Amplification of CRISPR plasmid vector containing gRNA sequence for TgPGK1 for pSAG1‐Cas9‐U6: sgPGK1-localization construction |
| gRNA-PGK1- loc‐Rv | AACTTGACATCCCCATTTAC |  |
| gRNA-PGK2-loc-Fw | GGTATATCAGCAAGGACCGCGTTTTAGAGCTAGAAATAGC | Amplification of CRISPR plasmid vector containing gRNA sequence for TgPGK2 for pSAG1‐Cas9‐U6: sgPGK2-localization construction |
| gRNA-PGK2-loc‐Rv | AACTTGACATCCCCATTTAC |  |
| gRNA-PGM1-loc-Fw | GCTAGAAGCAGACGGCAGCTGTTTTAGAGCTAGAAATAGC | Amplification of CRISPR plasmid vector containing gRNA sequence for TgPGM1 for pSAG1‐Cas9‐U6: sgPGM1-localization construction |
| gRNA-PGM1-loc‐Rv | AACTTGACATCCCCATTTAC |  |
| gRNA-PGM2-loc-Fw | GATGTGTGAGAATGTGCATGGTTTTAGAGCTAGAAATAGC | Amplification of CRISPR plasmid vector containing gRNA sequence for TgPGM2 for pSAG1‐Cas9‐U6: sgPGM2-localization construction |
| gRNA-PGM2-loc‐Rv | AACTTGACATCCCCATTTAC |  |
| gRNA-PGM3-loc-Fw | GCTCATTACAAAGAACTTGCGTTTTAGAGCTAGAAATAGC | Amplification of CRISPR plasmid vector containing gRNA sequence for TgPGM3 for pSAG1‐Cas9‐U6: sgPGM3-localization construction |
| gRNA-PGM3-loc‐Rv | AACTTGACATCCCCATTTAC |  |
| gRNA-PYK1-loc-Fw | GGAATCGAGTTTTTGGCAGTGTTTTAGAGCTAGAAATAGC | Amplification of CRISPR plasmid vector containing gRNA sequence for TgPYK1 for pSAG1‐Cas9‐U6: sgPYK1-localization construction |
| gRNA-PYK1-loc‐Rv | AACTTGACATCCCCATTTAC |  |
| gRNA-PYK2-loc-Fw | GACATCGAATGACAAACAGGGTTTTAGAGCTAGAAATAGC | Amplification of CRISPR plasmid vector containing gRNA sequence of TgPYK2 for pSAG1‐Cas9‐U6: sgPYK2-localization construction |
| gRNA-PYK2-loc‐Rv | AACTTGACATCCCCATTTAC |  |
| GPI-HA-loc-Fw | GCAGCTCGACGAGGAAGATTTTGGAGCACTACGTGCAGCAGTCGAAGGCTGGCTCGACGAGGATGTACCC | Amplification of homologous template from pSL24m-Linker-smFP-DHFR-LoxP-T7 for localization of TgGPI |
| GPI-HA-loc-Rv | ACGAAGACTGCAAACACCGCCGACCTAAACGAATAATGTAGAGCCACCCGCACCGCTTTCTCAACAGGAA |  |
| HK-HA-loc-Fw | TTGCGGATGACGGCTCTGGCAAGGGTGCTGCTCTGATCGCAGATGTGAACCGCGAGGTCCACACGAACCAGGA | Amplification of homologous template from pPUC19-Ty-3’UTR-DHFR for localization of TgHK |
| HK-HA-loc-Rv | GTATAAGCCATAGGACCGCTTCAGGAAATTCCGCGACGCTATGCGTGCGCGGAATTCATCCTGCAAGTGC |  |
| PFK1-HA-loc-Fw | CTGGTTAGGCATCGAGCAATTACGCCGCCCAACACTTCCAGGAGCGACGCGGCTCGACGAGGATGTACCC | Amplification of homologous template from pSL24m-Linker-smFP-DHFR-LoxP-T7 for localization of TgPFK1 |
| PFK1-HA-loc-Rv | GGACTGTGTGAGGTATTTCCCCCCCAAACTCGAATCGTCTTCTTCATCTTCACCGCTTTCTCAACAGGAA |  |
| PFK2-Ty-loc-Fw | AGGGCGCCATGAAAAGCGAAGCGTGCGGCGCGGCTGCATGCAGAGACCAACGCGAGGTCCACACGAACCAGGA | Amplification of homologous template from pPUC19-Ty-3’UTR-DHFR for localization of TgPFK2 |
| PFK2-Ty-loc-Rv | ACTCTTCTCGTCTGTCCCAGAGCCTCTGGTGTCGTCTCAGTTGTGCTCTGGGAATTCATCCTGCAAGTGC |  |
| TPI1-HA-loc-Fw | TGAAGAAGGACTTTCTCGACATTATTGCCTCTGGCATGAAGAAGAACGAGGGCTCGACGAGGATGTACCC | Amplification of homologous template from pSL24m-Linker-smFP-DHFR-LoxP-T7 for localization of TgTPI1 |
| TPI1-HA-loc-Rv | GGAAGCGCAAATACAACTGTCTTTTTCGTCAGCCTTCCCACCCGGTGTGCCACCGCTTTCTCAACAGGAA |  |
| TPI2-Ty-loc-Fw | CGCTGACAGGCGACTTTGTCTCCATCATTGATGCAGCGAAGCAGCAAGCCCGCGAGGTCCACACGAACCAGGA | Amplification of homologous template from pPUC19-Ty-3’UTR-DHFR for localization for localization of TgTPI2 |
| TPI2-Ty-loc-Rv | AGAGAGGCACAGCGAGGCGGGAAGCGTCGAGGTCTCTCAGTCGTGGGTAGGGAATTCATCCTGCAAGTGC |  |
| GAPDH1-HA-loc-Fw | CCAACCGCCTTGTTGAGCTGGCTCACTACATGTCCGTCCAGGACGGCGCGGGCTCGACGAGGATGTACCC | Amplification of homologous template from pSL24m-Linker-smFP-DHFR-LoxP-T7 for localization of TgGAPDH1 |
| GAPDH1-HA-loc-Rv | AAATGTGTGCTTACAGAATTTCCCAGGTCTTTCTCTACGAAATTATCTGCCACCGCTTTCTCAACAGGAA |  |
| GAPDH2-Ty-loc-Fw | CCAAAGACGGAGTTGTCAGCCCCGGCACCGGCCTCGACCGGAGACCCTTTCGCGAGGTCCACACGAACCAGGA | Amplification of homologous template from pPUC19-Ty-3’UTR-DHFR for localization for localization of TgGAPDH2 |
| GAPDH2-Ty-loc-Rv | TCACCTGTCTGCCTGGTGGAGCCATGCCTCCCGTCGCTTTTGCATGTATGGGAATTCATCCTGCAAGTGC |  |
| PGK1-HA-loc-Fw | AGCTCCTCGAAGGCAAAACTCTTCCTGGTGTCGCGGCTCTGTCCAACAAAGGCTCGACGAGGATGTACCC | Amplification of homologous template from pSL24m-Linker-smFP-DHFR-LoxP-T7 for localization of TgPGK1 |
| PGK1-HA-loc-Rv | AGTCCCCGGTAGCTCGTCACGTTATGCAAGCCACTTCCGGGGCGTTTCCACACCGCTTTCTCAACAGGAA |  |
| PGK2-Ty-loc-Fw | GCAAGACACTGCCAGGTGTTGCGGCGCTTTCAGACGAAGAAGAGACGCCTCGCGAGGTCCACACGAACCAGGA | Amplification of homologous template from pPUC19-Ty-3’UTR-DHFR for localization for localization of TgPGK2 |
| PGK2-Ty-loc-Rv | GAGGGCGCGCTGCTGTTTGCTCCACGCGGACGCACGTACGCCGTTTGCCCGGAATTCATCCTGCAAGTGC |  |
| PGM1-HA-loc-Fw | GAGAGTTTGGCGACGTAGGTCATCTGCAGGCGGATATGATAACTTACCACGGCTCGACGAGGATGTACCC | Amplification of homologous template from pSL24m-Linker-smFP-DHFR-LoxP-T7 for localization of TgPGM1 |
| PGM1-HA-loc-Rv | ACAAATTCTCCTTTTGAGAACGGCGCATCTGGCACAAAAAAACGTTAGTCCACCGCTTTCTCAACAGGAA |  |
| PGM2-Ty-loc-Fw | CGGAATTGAAGGCAAAGATGGAGGCTGTCGCCAACCAAGGAAAGGCCAAGCGCGAGGTCCACACGAACCAGGA | Amplification of homologous template from pPUC19-Ty-3’UTR-DHFR for localization for localization of TgPGM2 |
| PGM2-Ty-loc-Rv | AACAGATGGGCACCACTCGCGTTTCAGTCACGTGACTAGCCCCAAACCTGGGAATTCATCCTGCAAGTGC |  |
| PGM3-HA-loc-Fw | TACTTGAGGTTGACCGCGGGAAACAAGTACCAGCAACGATTGAATACGTGGGCTCGACGAGGATGTACCC | Amplification of homologous template from pSL24m-Linker-smFP-DHFR-LoxP-T7 for localization of TgPGM3 |
| PGM3-HA-loc-Rv | GAAATCGCTCCTAATGTGGCTGTTTTATGTGCAGTGTCAGTTAGACCGTCCACCGCTTTCTCAACAGGAA |  |
| PYK1-HA-loc-Fw | AAGAGGAAGTCGCTGGCTCCAGCAACCTTCTCAAGGTTCTTACTGTGGAGGGCTCGACGAGGATGTACCC | Amplification of homologous template from pSL24m-Linker-smFP-DHFR-LoxP-T7 for localization of TgPYK1 |
| PYK1-HA-loc-Rv | AAGGTGCTACTCGATTCTGGTTGGAGGGACATAAAAAAAAACCGTAACCACACCGCTTTCTCAACAGGAA |  |
| PYK2-Ty-loc-Fw | CGCGCCTCACCCGGCCAATTTTGACAGTGTGTACTCTCGAGTCAGGGCGACGCGAGGTCCACACGAACCAGGA | Amplification of homologous template from pPUC19-Ty-3’UTR-DHFR for localization for localization of TgPYK2 |
| PYK2-Ty-loc-Rv | CAGGAAGAACCTCGATACGCAACACCAGAATGCACGGACATATAACAGTGGGAATTCATCCTGCAAGTGC |  |
| gRNA-PGK2-Fw | GGAGTCCTGACCTCAAGCTGGTTTTAGAGCTAGAAATAGC | Amplification of CRISPR plasmid vector containing gRNA sequence for TgPGK2 for *Δpgk2* strain construction |
| gRNA-PGK2‐Rv | AACTTGACATCCCCATTTAC |  |
| gRNA-PYK2-Fw | GCCCGTTGCAAACTACCCAGGTTTTAGAGCTAGAAATAGC | Amplification of CRISPR plasmid vector containing gRNA sequences of TgPYK2 for *Δpgk2Δpyk2* strain construction |
| gRNA-PYK2‐Rv | AACTTGACATCCCCATTTAC |  |
| gRNA-PGK1-Fw | GCAGAAGATTGCGTGTCCTTGTTTTAGAGCTAGAAATAGC | Amplification of CRISPR plasmid vector containing gRNA sequences of TgPGK1 for TATi-iPGK1 strain construction |
| gRNA-PGK1‐Rv | AACTTGACATCCCCATTTAC |  |
| gRNA1-TPI1-Fw | GCATGACACCCAGTGCACCCGTTTTAGAGCTAGAAATAGC | Amplification of CRISPR plasmid vector containing gRNA1 sequences of TgTPI1 for DiCre-iTPI1 strain construction |
| gRNA1-TPI1‐Rv | AACTTGACATCCCCATTTAC |  |
| gRNA2-TPI1-Fw | GCTCAGTCACGTCAGGAATGGTTTTAGAGCTAGAAATAGC | Amplification of CRISPR plasmid vector containing gRNA2 sequences of TgTPI1 for DiCre-iTPI1 strain construction |
| gRNA2-TPI1‐Rv | AACTTGACATCCCCATTTAC |  |
| gRNA1-TPI2-Fw | CCGTCTCAGTCTGTTCCATCGTTTTAGAGCTAGAAATAGC | Amplification of CRISPR plasmid vector containing gRNA1 sequences of TgTPI2 for DiCre-iTPI2 strain construction |
| gRNA1-TPI2‐Rv | AACTTGACATCCCCATTTAC |  |
| gRNA2-TPI2-Fw | GGTCTCTCAGTCGTGGGTAGGTTTTAGAGCTAGAAATAGC | Amplification of CRISPR plasmid vector containing gRNA2 sequences of TgTPI2 for DiCre-iTPI2 strain construction |
| gRNA2-TPI2‐Rv | AACTTGACATCCCCATTTAC |  |
| gRNA1-GAPDH2-Fw | GCTACACCGTGGGGTGCGCCGTTTTAGAGCTAGAAATAGC | Amplification of CRISPR plasmid vector containing gRNA1 sequences of TgGAPDH2 for TATi-iGAPDH2 strain construction |
| gRNA1-GAPDH2‐Rv | AACTTGACATCCCCATTTAC |  |
| gRNA2-GAPDH2-Fw | GTCAAGTATTAAGCCAGCACGTTTTAGAGCTAGAAATAGC | Amplification of CRISPR plasmid vector containing gRNA2 sequences of TgGAPDH2 for TATi-iGAPDH2 strain construction |
| gRNA2-GAPDH2‐Rv | AACTTGACATCCCCATTTAC |  |
| pUC19-Fw | GGCGTAATCATGGTCATAGC | Amplification of pUC19 vector for pPYK1-DHFR and pPGK2::DHFR construction |
| pUC19-Rv | ATTCGCCCTATAGTGAGTCG |  |
| PGK2-5H-Fw | CGACTCACTATAGGGCGAATCGACAGGCACTATGGATAGC | Amplification of the 5′‐homology arm of PGK2  for pPGK2::DHFR construction |
| PGK2-5H-Rv | GATGTCTTCTGCGCGGGTTGGAAGGAGGAAACTGGCGTTG |  |
| PGK2-3H-Fw | GCCACAAGTTCAGCGTGTCCCGAGCTGTGCGTTAGAGTAG | Amplification of the 3′‐homology arm of PGK2  for pPGK2::DHFR construction |
| PGK2-3H-Rv | GCTATGACCATGATTACGCCGCGTAATGAACATGGGTTCG |  |
| Loxp-DHFR-loxp-Fw | CAACCCGCGCAGAAGACATC | Amplification of Loxp-DHFR-Loxp fragments for pPGK2::DHFR construction |
| Loxp-DHFR-loxp-Rv | GGACACGCTGAACTTGTGGC |  |
| Vec-5H-CAT-3H-Fw | CTATCTGTTGACTCGTGACG | Amplification of pPYK2::DHFR for pPYK2::CAT construction |
| Vec-5H-CAT-3H-Rv | CCTGTGAGAAGAGGGAACAT |  |
| iPGK1-5H-Fw | CGACTCACTATAGGGCGAATGAAGGCAGCAAACAGCTACC | Amplification of the 5′‐homology arm of PGK1 for pPGK1-teto7 construction |
| iPGK1-5H-Rv | CTCACGGGATTTACAGCCTGGGGAATCCCAGAAACTGACT |  |
| iPGK1-3H-Fw | CGAACCAGGACCCGCTCGATCTGGCAAACAAGCTCGGAAT | Amplification of the 3′‐homology arm of PGK2 for pPGK1-teto7 construction |
| iPGK1-3H-Rv | GCTATGACCATGATTACGCCCCTTCATCGGGACGTTGAAG |  |
| DHFR‐Fw | CAGGCTGTAAATCCCGTGAG | Amplification of DHFR-Tet-Ty fragment for pPGK1-DHFR construction |
| Tet-Ty-R | ATCGAGCGGGTCCTGGTTCG |  |
| iTPI1-CDS-Fw | AGTTATAGAATTCCGACAAAATGGTGCGCACCCCGTGGGT | To amplify the CDS of TPI1 for pDiCre-TPI1 construction |
| iTPI1-CDS-Rv | TCCTGGTTCGTGTGGACCTCCTCGTTCTTCTTCATGCCAG |  |
| iTPI2-CDS-Fw | AGTTATAGAATTCCGACAAAATGTCGGGCTCCGCTTCCTC | Amplification of the CDS of TPI2 for pDiCre-TPI2 construction |
| iTPI2-CDS-Rv | TCCTGGTTCGTGTGGACCTCGGCTTGCTGCTTCGCTGCAT |  |
| pDiCre-Fw | GAGGTCCACACGAACCAG | Amplification of the vector for pDiCre-TPI1 or pDiCre-TPI2 construction |
| pDiCre-Rv | TTTGTCGGAATTCTATAA |  |
| iGAPDH2-5H-Fw | TACTGAGAGTGCACCATATGGAGTCTGACAGCTGTGTAG | Amplification of the 5′‐homology arm of GAPDH2 for pGAPDH2-teto7 construction |
| iGAPDH2-5H-Rv | CTCACGGGATTTACAGCCTGGGGGTTCACACGATTCAGTC |  |
| iGAPDH2-3H-Fw | ACCAGGACCCGCTCGATTAAAGACGCGGGAGACTCCTGTT | Amplification of the 3′‐homology arm of GAPDH2 for pGAPDH2-teto7 construction |
| iGAPDH2-3H-Rv | CTAGAGGATCCCCGGGTACCGTCTCTGAAGTGTAGCGGTC |  |
| iGAPDH2-full-length-Fw | GAATTCCCTTTTTCGACAAAATGTCTCTGTATCCGAGATC | Amplification of the full-length GAPDH2 for pGAPDH2-teto7 construction |
| iGAPDH2-full-length-Rv | TTAATCGAGCGGGTCCTGGTTCGTGTGGACCTCAAAGGGTCTCCGGTCGAGGC |  |
| DHFR-Teto7-F | CAGGCTGTAAATCCCGTGAG | Amplification of the DHFR-Teto7 fragment for pGAPDH2-teto7 construction |
| DHFR-Teto7-R | TTTGTCGAAAAAGGGAATTC |  |
| pUC19-TATi-Fw | CATATGGTGCACTCTCAGTA | Amplification of the vector for pPGK1-teto7 and pGAPDH2-teto7 construction |
| pUC19-TATi-Rw | GGTACCCGGGGATCCTCTAG |  |
| MVA-Fw | TTTTCTAGAATTCCGACAAAATGACGAAGAAGGTGGGCGT | Amplification of the CDS of MVA for pcompMVA-HA-HX-UPRT construction |
| MVA-Rw | TCTGGAACATCGTAAGGATAAGGGTTCGCGTTGGGGTTGG |  |
| Vector-HA-HX-Fw | TATCCTTACGATGTTCCAGA | Amplification of the vector for pcompMVA-HA-HX-UPRT construction |
| Vector-HA-HX-Rw | TTTGTCGGAATTCTAGAAAAAATGCCAACGAGTA |  |
| CAT-Fw | ATGTTCCCTCTTCTCACAGGGATATCGAATTCCTGCAGCC | To produce the CAT fragment for pPYK2::CAT construction |
| CAT-Rv | CCGTCACGAGTCAACAGATAGCTCTAGAACTAGTGGATCC |  |
| PGK2-truncation-SUMO-Fw | CCGCGAACAGATTGGAGGTCTGGGTCAGAGCAGGTTGTC | Amplification of PGK2 truncated fragment from RH cDNA for pET-28a-PGK2-truncation construction |
| PGK2-truncation-SUMO-Rv | TCGAATTCGGATCCTCTAGTTTCAAGGAGCAGAATGTCGC |  |
| PGK1-full-length-SUMO-Fw | CTCACAGAGAACAGATTGGTATGCTGGCAAACAAGCTCGG | Amplification of PGK1-full-length fragment from RH cDNA for pET-28a-2HIS-SUMO-PGK1 construction |
| PGK1-full-length-SUMO-Rv | CAGTGGTGGTGGTGGTGGTGTTTGTTGGACAGAGCCGCGA |  |
| PGK2-full-length-SUMO-Fw | CTCACAGAGAACAGATTGGTGTCTCGTTTTCGTCGCCGTC | Amplification of PGK2-full-length fragment from RH cDNA for pET-28a-2HIS-SUMO-PGK2 construction |
| PGK2-full-length-SUMO-Rv | CAGTGGTGGTGGTGGTGGTGAGGCGTCTCTTCTTCGTCTG |  |
| SUMO-vec-Fw | ACTAGAGGATCCGAATTCGA | Amplification of SUMO linearized vector for pET-28a-PGK2-truncation construction |
| SUMO-vec-Rv | ACCTCCAATCTGTTCGCGG |  |
| 2HIS-SUMO-Fw | caccaccaccaccaccactg | Amplification of SUMO linearized vector for pET-28a-2HIS-SUMO-PGK1 and pET-28a-2HIS-SUMO-PGK2 construction |
| 2HIS-SUMO-Rv | accaatctgttctctgtgag |  |
| 5’-UpU5PYK2-Fw | CTGTCGTCATCAGACTGGAG | PCR1 of *∆pyk2∆pgk2* strain |
| 3'-in-CAT-Rv | CTTCCTACAGGTGCACATTG |  |
| 5'-in-CAT-Fw | CTGGGTCCGAATCTCTGAAC | PCR2 of *∆pyk2∆pgk2* strain |
| 3’-DnU3PYK2-Rv | GATGACCCTTCTCAGGCAAC |  |
| In-PYK2-Fw | GGCGAGCTGGGATTATGAAG | PCR3 of *∆pyk2∆pgk2* strain |
| In-PYK2-Rw | CCTTCTCTAAGTGCCGTACG |  |
| 5’-UpU5PGK2-Fw | CACGGTTATACCCTGAGCAC | PCR1 of *∆pgk2* strain |
| 3’-InDHFR-Rv | CGGGAAAGTCACGCATATGG |  |
| 5’-InDHFR-Fw | CGCACGGACGAATCCAGATG | PCR2 of *∆pgk2* strain |
| 3’-DnU3PGK2-Rv | CATATTGGGCTGGTTCGGTC |  |
| In-PGK2-Fw | GATTCGGAGGATCTCCCAAC | PCR3 of *∆pgk2* strain |
| In-PGK2-Rw | GGATACAGAGGGCTCTTAGC |  |
| 5’-UpU5PGK1-Fw | TCTTTCCCGTCGCAAGTGTG | PCR1 of TATi-iPGK1 strain |
| 3’-InDHFR-Rv | CTCAGTCAGGCAGTCTCCCT |  |
| 3’-DnU3PGK1-Rv | TTGCCTTCCTCTTCGATGTG | PCR2 of TATi-iPGK1 strain |
| 5’-InDHFR-Fw | GCTGTAGTCACTGCTGATTC |  |
| In-PGK1-Fw | CCTGCGCTTCCTCATTTGAC | PCR3 of TATi-iPGK1 strain |
| In-PGK1-Rw | ACGGGTGATGGTTTCTGGTC |  |
| TPI1-PCR1-Fw | ATGTGACAACCGCAAGAACC | PCR1 of DiCre-iTPI1 strain |
| TPI1-PCR1-Rv | ATCACCAACCGCACCCAATG |  |
| TPI1-PCR2-Fw | TGGCTGTCTAGCGGAAATAC | PCR2 of DiCre-iTPI1 strain |
| TPI1-PCR2-Rv | CTGGGCACTTCTACTGAAAC |  |
| TPI1-PCR3-Fw | GGCAACTGGAAGTGCAATGG | PCR3 of DiCre-iTPI1 strain |
| TPI1-PCR3-Rv | CGCCAACAAGGAAACCATCG |  |
| TPI2-PCR1-Fw | CCGTCGTTCCTCCAGCATTC | PCR1 of DiCre-iTPI2 strain |
| TPI2-PCR1-Rv | ATCACCAACCGCACCCAATG |  |
| TPI2-PCR2-Fw | TGGCTGTCTAGCGGAAATAC | PCR2 of DiCre-iTPI2 strain |
| TPI2-PCR2-Rv | GATCTGGCGTAGAAACACTG |  |
| TPI2-PCR3-Fw | ATGTCGGGCTCCGCTTCCTC | PCR3 of DiCre-iTPI2 strain |
| TPI2-PCR3-Rv | GGCTTGCTGCTTCGCTGCAT |  |
| compMVA-PCR1-Fw | ATGACGAAGAAGGTGGGCGT | PCR1 of DiCre-iTPI2compMVA strain |
| compMVA-PCR1-Rv | AGGGTTCGCGTTGGGGTTGG |  |
| compMVA-PCR2-Fw | GGGTGCCTACGTTCTTCTAC | PCR2 of DiCre-iTPI2compMVA strain |
| compMVA-PCR2-Rv | GTCCCCAGGTAGCGAGAACG |  |
| GAPDH2-PCR1-Fw | ACTAGTACTACCGTACGAGC | PCR1 of TATi-iGAPDH2 strain |
| GAPDH2-PCR1-Rv | CTCAGTCAGGCAGTCTCCCT |  |
| GAPDH2-PCR2-Fw | GTCCACACGAACCAGGACCC | PCR2 of TATi-iGAPDH2 strain |
| GAPDH2-PCR2-Rv | CTGTGTATCGCTGTTGACCT |  |
| GAPDH2-PCR3-Fw | CCACGTCTCCGGCTAAAAGT | PCR3 of TATi-iGAPDH2 strain |
| GAPDH2-PCR3-Rv | CACAGGAACGCACAGAGCTG |  |
| TPI2-qPCR-Fw | CGCCTCCGAGTCTCTTCATC | qPCR for the detection of TPI2 expression |
| TPI2-qPCR-Rv | TTCTTCGCCACGACTTGGTT |  |
| GAPDH2-qPCR-Fw | ACTACGTCTGCGAATCCACTG | qPCR for the detection of GAPDH2 expression |
| GAPDH2-qPCR-Rv | CCAGGTCTGTTGACGTGCTT |  |
| tubulin-qPCR-Fw | GGTACACGGGTGAAGGT | qPCR for the detection of beta-tubulin expression |
| tubulin-qPCR-Rv | ATTCTCCCTCTTCCTCTGCG |  |
