# Supplementary material for: Two apicoplast dwelling glycolytic enzymes provide key substrates for metabolic pathways in the apicoplast and are critical for Toxoplasma growth
Source: PLoS Pathog. 2022 Nov 30;18(11):e1011009. doi: 10.1371/journal.ppat.1011009 (PMC9744290; doi:10.1371/journal.ppat.1011009)
Supplement: S3 Table — (DOCX) [file ppat.1011009.s008.docx]

**Table S3. Raw data of DOXP/MEP/IPP/DMAPP levels measured by MS.**

|  | iTPI2-rapa | | | iTPI2+rapa | | |
| --- | --- | --- | --- | --- | --- | --- |
|  | Exp 1 | Exp 2 | Exp 3 | Exp 1 | Exp 2 | Exp 3 |
| IPP/DMAPP | 3.42×10^4^ | 3.88×10^4^ | 2.36×10^4^ | 1.12×10^4^ | 1.27×10^4^ | 6.82×10^3^ |
| DOXP | 9.39×10^3^ | 1.05×10^4^ | 9.93×10^3^ | 3.90×10^3^ | 7.16×10^3^ | 5.95×10^3^ |
| MEP | 4.26×10^4^ | 3.69×10^4^ | 4.07×10^4^ | 1.07×10^4^ | 1.42×10^4^ | 2.08×10^4^ |

|  | iTPI1-rapa | | | iTPI1+rapa | | |
| --- | --- | --- | --- | --- | --- | --- |
|  | Exp 1 | Exp 2 | Exp 3 | Exp 1 | Exp 2 | Exp 3 |
| IPP/DMAPP | 4.47×10^4^ | 3.29×10^4^ | 1.85×10^4^ | 5.72×10^4^ | 9.24×10^4^ | 3.95×10^4^ |
| DOXP | 1.67×10^4^ | 1.95×10^4^ | 1.24×10^4^ | 4.35×10^4^ | 6.11×10^4^ | 5.07×10^4^ |
| MEP | 1.47×10^5^ | 1.72×10^5^ | 1.09×10^5^ | 3.31×10^5^ | 4.60×10^5^ | 4.96×10^5^ |

|  | iTPI2comMVA+Rapa -MEV | | | iTPI2comMVA+Rapa+MEV | | |
| --- | --- | --- | --- | --- | --- | --- |
|  | Exp 1 | Exp 2 | Exp 3 | Exp 1 | Exp 2 | Exp 3 |
| IPP/DMAPP | 1.38×10^4^ | 3.72×10^3^ | 1.14×10^4^ | 6.00×10^5^ | 1.46×10^5^ | 6.01×10^5^ |
| DOXP | 6.50×10^3^ | 1.54×10^4^ | 1.40×10^4^ | 9.60×10^3^ | 1.17×10^4^ | 1.39×10^4^ |
| MEP | 4.72×10^4^ | 3.50×10^4^ | 5.03×10^4^ | 3.85×10^4^ | 4.18×10^4^ | 4.15×10^4^ |

|  | iGAPDH2-ATc | | | iGAPDH2+ATc | | |
| --- | --- | --- | --- | --- | --- | --- |
|  | Exp 1 | Exp 2 | Exp 3 | Exp 1 | Exp 2 | Exp 3 |
| IPP/DMAPP | 9.22×10^3^ | 8.90×10^3^ | 3.29×10^4^ | 2.57×10^3^ | 1.23×10^3^ | 8.35×10^3^ |
| DOXP | 1.95×10^4^ | 2.59×10^4^ | 1.86×10^4^ | 1.59×10^4^ | 2.26×10^4^ | 3.03×10^4^ |
| MEP | 9.79×10^4^ | 1.15×10^5^ | 1.32×10^5^ | 4.34×10^4^ | 5.55×10^4^ | 7.61×10^4^ |
